# Causal Association of Golgi Protein 73 With Coronary Artery Disease: Evidence from Proteomics and Mendelian Randomization

**DOI:** 10.7150/ijms.94179

**Published:** 2024-08-12

**Authors:** Yi-Fen Lin, Li-Zhen Liao, Shu-Yi Wang, Shao-Zhao Zhang, Xiang-Bin Zhong, Hui-Min Zhou, Xing-Feng Xu, Zhen-Yu Xiong, Yi-Quan Huang, Meng-Hui Liu, Yue Guo, Xin-Xue Liao, Xiao-Dong Zhuang

**Affiliations:** 1 Cardiology Department, the First Affiliated Hospital, Sun Yat-Sen University, Guangzhou, Guangdong China.; 2 NHC Key Laboratory of Assisted Circulation (Sun Yat-Sen University), Guangzhou, Guangdong China.; 3 Guangdong Engineering Research Center for Light and Health, Guangdong Pharmaceutical University, Guangzhou Higher Education Mega Center, Guangzhou, Guangdong, China.; 4 Department of Rheumatology, the First Affiliated Hospital, Sun Yat-Sen University, Guangzhou, Guangdong China.

**Keywords:** Coronary artery disease, Golgi protein 73, Mendelian randomization

## Abstract

**Background:** Identification of the unknown pathogenic factor driving atherosclerosis not only enhances the development of disease biomarkers but also facilitates the discovery of new therapeutic targets, thus contributing to the improved management of coronary artery disease (CAD). We aimed to identify causative protein biomarkers in CAD etiology based on proteomics and 2-sample Mendelian randomization (MR) design.

**Methods:** Serum samples from 33 first-onset CAD patients and 31 non-CAD controls were collected and detected using protein array. Differentially expressed analyses were used to identify candidate proteins for causal inference. We used 2-sample MR to detect the causal associations between the candidate proteins and CAD. Network MR was performed to explore whether metabolic risk factors for CAD mediated the risk of identified protein. Vascular expression of candidate protein *in situ* was also detected.

**Results:** Among the differentially expressed proteins identified utilizing proteomics, we found that circulating Golgi protein 73 (GP73) was causally associated with incident CAD and other atherosclerotic events sharing similar etiology. Network MR approach showed low-density lipoprotein cholesterol and glycated hemoglobin serve as mediators in the causal pathway, transmitting 42.1% and 8.7% effects from GP73 to CAD, respectively. Apart from the circulating form of GP73, both mouse model and human specimens imply that vascular GP73 expression was also upregulated in atherosclerotic lesions and concomitant with markers of macrophage and phenotypic switching of vascular smooth muscle cells (VSMCs).

**Conclusions:** Our study supported GP73 as a biomarker and causative for CAD. GP73 may involve in CAD pathogenesis mainly via dyslipidemia and hyperglycemia, which may enrich the etiological information and suggest future research direction on CAD.

## Introduction

Coronary artery disease (CAD) is the most common cardiovascular disease globally, contributing to over 9.5 million deaths annually and posing a significant burden on public health 1. The nationwide longitudinal Swedish SWEDEHEART registry presented a substantial reduction in 1-year mortality of myocardial infarction (MI) from 1995 to 2014, with the gradual widespread implementation of new evidence-based treatment strategies including reperfusion, primary percutaneous coronary intervention, dual antiplatelet therapy, statins, beta-blockers, and angiotensin-converting-enzyme/angiotensin-2-receptor inhibitors 2, 3. However, the improvement effects appear to reach a plateau, and no notable improvement has been observed over the last decade 4. Such a dilemma indicated residual risk in CAD onset and a limited understanding of the pathogenic mechanisms driving atherosclerosis 4. Although traditional risk factors for CAD were well-established, the underlying mechanism behind these risk factors or whether additional pathways bypassing known risk factors remains incompletely understood 5, 6. Hence, further elucidation of the molecular mechanism may guide more effective strategies for interfering with the initiation and progression of atherosclerosis.

Serum proteins serve critical roles in numerous disease processes and provide an essential source of therapeutic targets 7, 8. The advent of high-throughput proteomic technology enables the comprehensive monitoring of each individual's proteomic landscapes, thus discovering novel disease biomarkers and understanding the pathophysiological network underlying the disease, thereby providing new therapeutic candidate targets 9. However, given the nature of the observational study, bioinformatics analysis based on protein arrays is inadequate to infer causality due to multiple limitations, such as selection bias, potential confounding, and reverse causation 10. Mendelian Randomization (MR), an emerging epidemiological methodology in causal inference, provides alternative opportunities to assess the causality of biomarkers in disease onset. Essentially, the MR approach use randomly allocated genetic variants as instruments, which were built preceding the onset of disease thus avoiding the confounders theoretically, to evaluate the causal association from exposure factors to outcome unbiasedly 11.

In recent years, large-scale genome-wide association studies (GWAS) have mapped the genetic variations associated with circulating protein profiles, greatly facilitating the causality assessment of proteome in disease etiology using the MR approach 12, 13. Here, by leveraging proteomics data from a real-world cohort and large-scale GWAS on the circulating proteome, we aimed to comprehensively screen the differentially expressed proteins (DEPs) in CAD patients using protein array and identify causal biomarkers in CAD etiology based on a 2-sample MR study. For the identified circulating protein, we also investigated the association of its vascular expression *in situ* with atherosclerosis.

## Methods

### Study Design

The study consisted of seven steps (Figure S1): (1) Measurements of 640 proteins using proteomic technology in 33 CAD patients and 31 controls; (2) Screening for DEPs between CAD and control individuals; (3) Exploring the causal association between candidate proteins with CAD using 2-sample MR; (4) Sensitivity analyses to validate the association between the identified protein with CAD; (5) Exploring the causal association between the identified protein with other atherosclerosis diseases sharing the same etiology; (6) Exploring the mediating role of metabolic risk factors in the pathogenic pathway from GP73 to CAD using network MR; (7) Investigating the association between vascular expression of the identified protein *in situ* with atherosclerosis.

### Study population

The participants were enrolled from the REal-world Data of CARdiometabolic ProtEcTion (RED-CARPET, ChiCTR2000039901) study at the First Affiliated Hospital of Sun Yat-Sen University (Detail in Text S1). Studies on human specimens followed the Declaration of Helsinki guidelines. We recruited 64 individuals (aged 34 to 80 years) who were admitted due to chest distress or pain and received coronary angiography examinations from January 2017 to February 2018. Demographics, lifestyle factors, physical measurements, clinical history, and laboratory data of participants were collected by trained staff (Detail in Text S1). Gensini score system was used to evaluate the severity of coronary artery stenosis 14. CAD was defined as Gensini score > 0 with greater than 50% stenosis in any coronary arteries. Those with self-reported chest distress or pain without any coronary stenosis proven by coronary angiography were included in control group.

### Blood sampling and proteomics measurements

Blood samples for proteomics were drawn in the morning after fasting for at least 10 hours. The blood samples were centrifuged at 4,000 rpm/min for 10 minutes after resting for 45 minutes. Serum samples were aliquot and stored at -80 °C until assays for proteomics. Relative expression levels of 640 human cytokines were measured using G-Series Human Cytokine Array 640 Kit. (RayBiotech, Inc.) according to the manufacturer's protocol (Text S2). Based on sandwich-based ELISA, the signals of the cytokine-antibody-biotin complex were then detected by an Axon GenePix laser scanner. RayBio Q Analyzer tool was used to analyze the data.

### Publicly available GWAS summary data for 2-sample MR analyses

We obtained association summary statistics for candidate proteins from the published GWAS, the Age, Gene/Environment Susceptibility (AGES)-Reykjavik study 13, 15. The AGES study measured 4,782 serum protein concentrations based on the SOMAscan platform and detected 54,469 genetic variants from the HumanExome BeadChip exome array in 5,343 participants. A total of 2,019 protein quantitative trait loci (pQTL) were independently associated with circulating levels of 2,135 serum proteins at a Bonferroni corrected P-value threshold 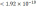 (0.05/54,469/4,782) 13. Assay details of the AGES study have been previously described {#14}.

Effect size and standard errors of genetic instruments on CAD were extracted from CARDIoGRAMplusC4D 1,000 Genomes-based GWAS, one of the largest GWAS on CAD comprising 60,801 cases and 123,504 controls mainly from European ancestry 16. CAD was defined as an inclusive diagnosis of myocardial infarction, acute coronary syndrome, chronic stable angina, or coronary stenosis of >50%. The extent of sample overlap between exposure and outcome samples seemed to be low because they were derived from different consortiums. Details and web links for downloading the data of other GWAS used for analysis were summarized in Table S1.

### Selection of genetic instruments

For each candidate protein identified through proteomics analysis, we selected pQTLs associated with their serum concentrations at a Bonferroni-corrected P-value threshold from the AGES study (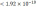) if available. When two or more pQTLs associated with a particular protein were located at the same chromosome, we evaluated the correlations between the pQTLs using LDlink Tool (http://ldlink.nci.nih.gov/). Those independent pQTLs, defined as linkage disequilibrium 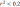 within 500 kb with a reference panel consisting of European populations, were retained for subsequent analysis 17, 18. We used the F-statistic to assess the strengths of the pQTLs based on an equation developed by Bowden et al.: 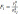, while 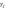 and 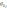 refer to the estimate and standard deviation of the association between pQTLs and proteins, respectively 19. pQTLs with an F-statistic>10 and minor allele frequency (MAF) > 0.001 were selected as genetic instrument variables (IVs) 20. The F-statistic for 14 pQTLs ranged from 40.79 to 5575.4, reaching the threshold of F-statistic >10, typically recommended for MR analyses (Table S2) 20. For those pQTLs absent in GWAS on the outcome, highly correlated proxy pQTLs (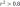) were used when available. All pQTLs of candidate proteins used in primary MR analysis were listed in Table S2.

### Differential expression analysis

Chip background adjustments and inter-chip normalization on row data were performed using Raybiotech software. The normalized data were subjected to differentially expressed analysis based on the 'limma' R package (version 3.48.3) 21. The log_2_-fold change (log2FC) was calculated as the logarithm base 2 of the ratio of protein expression levels in individuals with CAD (CAD group) to those without CAD (non-CAD group), represented as log_2_ (CAD/non-CAD). DEPs were defined as P-value<0.05 and |log_2_FC|>0.263 (equivalent to FC>1.2 or FC< 0.83) 22, where FC>1.2 indicated up-regulated DEPs and FC<0.83 indicated down-regulated DEPs. Volcano Plot ('ggplot2' package) was generated to visualize the DEPs. The DEPs were used for subsequent causal inference based on a 2-sample MR approach.

### Two-sample Mendelian Randomization

In primary MR analysis to assess the causal effects of the candidate proteins on CAD, we performed the Wald Ratio method for those with a single IV and the inverse variance weighted (IVW) method based on the fixed-effect (FE) model for those with two or more IVs. A random-effect (RE) model was used in the IVW method if heterogeneity across pQTLs exists. The odds ratio (OR) and 95% confidence interval (CI) of the causal relationship were estimated based on the predicted beta coefficient and standard error. A Benjamini-Hochberg false discovery rate (FDR) of < 0.05 indicated a causal relationship for adjusting multiple comparisons. For the identified proteins, the causal effects of genetic instruments on outcomes were summarized in Table S3.

For protein reaching FDR<0.05, we further applied several MR methods to validate the robustness of the causality as sensitivity analysis. The weighted median method can provide reliable estimates of causal relationships when at least half of the IVs are valid, even with horizontal pleiotropy 23. Based on the assumption that the most common causal effect is consistent with the real causal effect, the weighted mode method usually has a low type 1 error rate inflation 24, 25. MR-Egger regression assessed the association between exposure and outcome by performing a weighted linear regression of the pQTLs-outcome estimates on pQTLs-exposure estimates 26. The leave-one-out approach removed IVs in turn to assess the effect of outlying IVs. We also conducted the Mendelian randomization pleiotropy residual sum and outlier (MR-PRESSO) test to investigate and correct outliers of IVs with pleiotropic effects 27. Cochran's Q test evaluated the heterogeneity of the causal effect across IVs on the outcome. MR-Egger regression was also used to detect pleiotropy. The forest plot depicted the predicted effect and standard error of individual IVs on CAD, while the scatter plot displayed the relationship between the IVs with candidate proteins and CAD. The IV's estimate against its precision was plotted in the funnel plot, where asymmetry implied directional horizontal pleiotropy.

Other sensitivity analyses included external validation based on additional GWAS data and assessing the causality with other atherosclerosis diseases, including myocardial infarction, ischemic stroke, and its subtype, peripheral artery disease (PAD) 16, 28-30. Post-hoc power calculation was performed based on mRnd (http://cnsgenomics.com/shiny/mRnd/) 31. This study is reported as per the Strengthening the Reporting of MR studies (STROBE-MR) guideline (Text S3) 31.

### Network Mendelian randomization and mediation analysis

We investigate the role of metabolic risk factors in the causal pathway from identified proteins to CAD using the network MR approach 33, 34, including the following traits: lipid profiles 35, 36 [low-density lipoprotein cholesterol (LDL-c), high-density lipoprotein cholesterol (HDL-c), total cholesterol (TC)], glycemic profile 29, 37, 38 [glycated hemoglobin (HbA1c), homeostasis model assessment of β-cell function (HOMA-β), homeostatic model assessment for insulin resistance (HOMA-IR)]. Network MR analyses were conducted to identify potential metabolic mediators in the causal pathway (methodology detailed in Figure S2), and mediation analysis was used to quantify the proportion of effects mediated by the investigated mediator (Text S4) 33, 39.

### Vascular GP73 expression and atherosclerosis

For the identified protein (Golgi protein 73, GP73), we investigate its vascular expression in arteries with different atherosclerotic states. Apolipoprotein-E-gene-deficient (ApoE-/-) mice (8-week old, male, n=5) were fed with a high-fat diet (ApoE-HFD) for 12 weeks to establish the atherosclerosis model, while 5 ApoE-/- mice were fed with normal diet (ApoE-ND) as control. (1) Western blot analysis: Total proteins were extracted from the isolated abdominal aortic tissues using standard procedures. Western blot was performed with specific antibodies against GP73 (1:5000, Proteintech) and β-actin (1:5000, Abcam). An enhanced chemiluminescence reagent kit (Applygen Technologies, Beijing, China) was used to visualize the protein, and Image J software (NIH, Bethesda, MD, USA) was used to quantify band intensity. (2) Immunofluorescence Analysis: Immunofluorescence staining method was implemented as previously described 40. Frozen aortic root sections from ApoE-HFD and ApoE-ND mice were double-stained with anti-GP73 (rabbit, Proteintech) antibodies and macrophage marker, anti-F4-80 (rat; Abcam) antibodies. The slides were imaged by a confocal laser scanning microscope (LSM780, Zeiss, Oberkochen, Germany). Other information on experimental procedures was provided in Text S5. The animal experiment was approved by the ethics committees of Sun Yat-sen University (Approval NO. SYSU-IACUC-2023-000052). Public transcriptome data of human artery specimens were downloaded from GEO (Gene Expression Omnibus) website (http://www.ncbi.nlm.nih.gov/geo/). Differential expressed analyses were utilized to compare vascular GP73 expression between atherosclerotic artery (n=69) with control artery (n=35) (GSE100927), and between early (n=13) with advanced plaques (n=16) on human carotid (GSE28829) (Table S4) 41, 42. Associations between GP73 mRNA expression level and other atherosclerotic markers in plaque were determined via Pearson correlations.

### Statistical analysis

Statistical analyses were performed in R version 4.0.1 and GraphPad Prism (version 7.0). Statistical comparison between two groups was based on Student's t test (continuous variables), χ^2^ tests (categorical variables), and the Kruskal-Wallis test as appropriate. Pearson correlation was used to determine the linear relationship between continuous variables. MR analyses were conducted using the 'TwoSampleMR' and 'MR-PRESSO' R package. 'DESeq2' R package was used for differential expressed analysis. A P-value < 0.05 was considered statistically significant unless specifically indicated otherwise.

## Results

### Differentially expressed analysis

**Figure 1:**
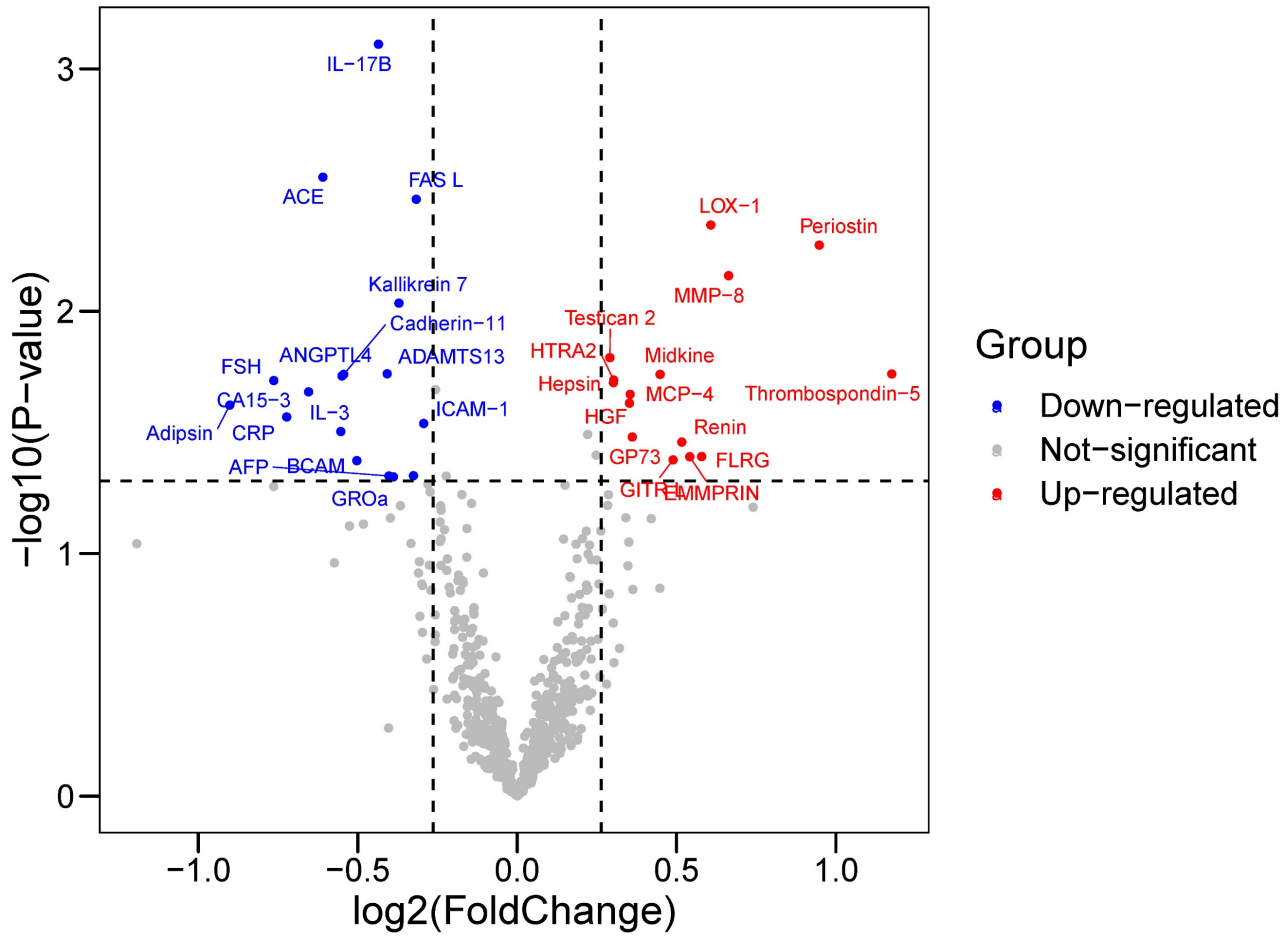
** Volcano Plot showed the differentially expressed proteins (DEPs) between CAD and control groups.** Differentially expressed proteins were identified between 33 CAD and 31 controls. The x-axis and y-axis correspond to log_2_(Foldchange) value and -log_10_ (P-value), respectively. Circulating proteins with |log_2_(Foldchange)|>0.263 and P-value <0.05 were considered as significantly differentially expressed. The red dots represent significantly up-regulated proteins, while blue dots display significantly down-regulated proteins in CAD group.

Baseline characteristics of 64 participants (33 in the CAD group and 31 in the control group) enrolled in proteomics were shown in Table 1. Cardiovascular risk factors showed similar distributions in the two groups. Individuals in the CAD group exhibits higher Gensini score and elevated concentration of creatine kinase-MB and cardiac troponin T. Among 640 proteins detected, we identified 33 DEPs (|log_2_FC|>0.263, P-value<0.05) in the CAD groups compared with the control group (Table S5), among which 15 were upregulated and 17 were downregulated (Figure 1). Some proteins were already well known in CAD pathogenesis, e.g., renin; however, there were still some proteins not yet been investigated in the field of CAD.

### Genetically determined circulating levels of candidate proteins and risk of CAD

Among the DEPs identified by protein array, genetic IVs for 17 DEPs were extracted from the AGES study. In a 2-sample MR analysis using the IVW method, genetically predicted higher GP73 concentration was positively associated with increased CAD risk (OR, 1.11; 95%CI, 1.05-1.18; p-value<0.001; Table S6) after accounting for multiple comparisons (FDR=0.005). However, we detected no evidence of a causal relationship between genetically determined levels of the other 16 DEPs with CAD risk (Table S6). In a post-hoc power calculation for GP73, the proportion of variance in the GP73 level explained by genetic instruments was 11%. Assuming the real causal OR of GP73 on CAD was 1.11, we had sufficient statistical power (>80%) to detect the causal association between GP73 and CAD with a total sample size of 184,305 (33.0% CAD cases) and the significance level α of 0.05, even at an α of 0.01 for multi-comparison.

**Figure 2:**
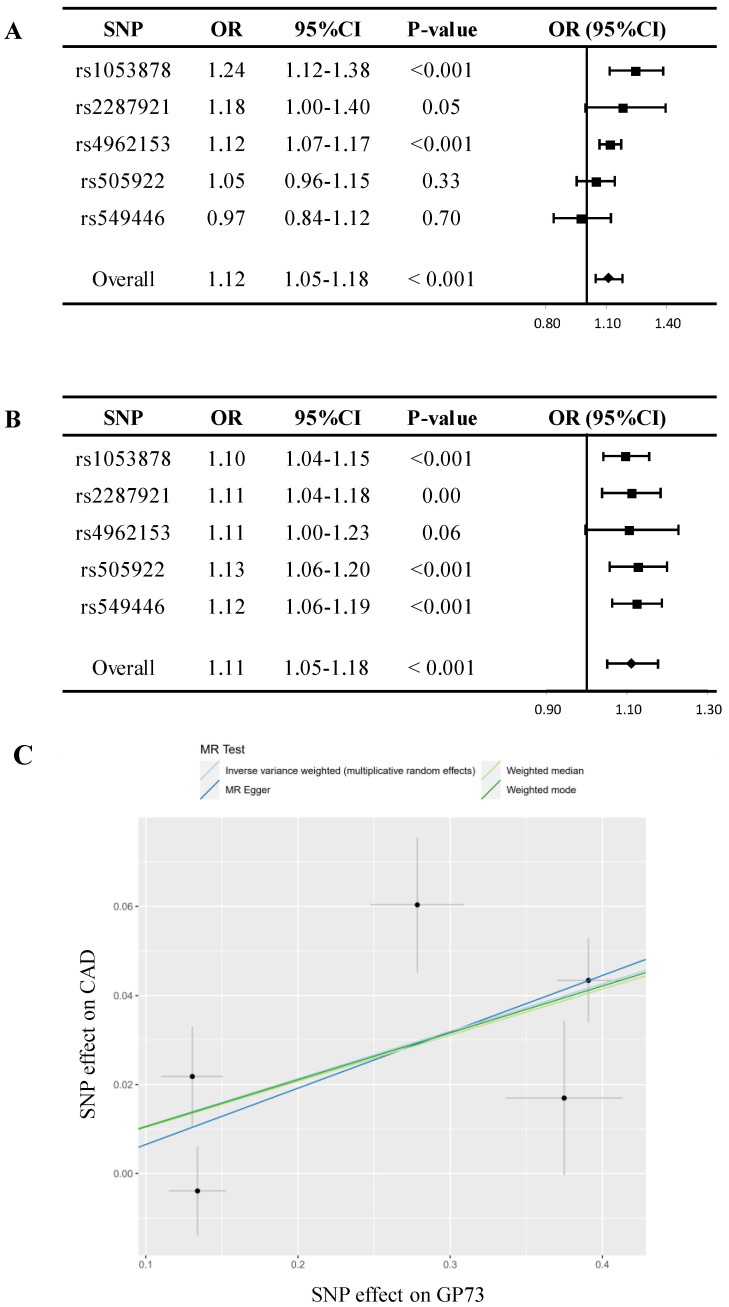
** Mendelian randomization analysis for circulating GP73 level and risk of CAD. (A)** Forest plot displays the summary of MR estimates on coronary artery disease (CAD) using each protein quantitative trait loci (pQTL) as instrument via the Wald method. The overall OR of GP73 on CAD is estimated based on the inverse variance weighted (IVW) method. **(B)** Leave-one-out analysis excludes one pQTL at a time and test the MR estimate from remaining pQTLs. Five pQTLs showed consistent results and reported no outlier pQTL. **(C)** Scatter plots showed the dose-response relationship between circulating GP73 level and CAD risk.

In sensitivity analyses, despite MR estimate using MR Egger did not detect a causal relationship between GP73 concentrations with CAD, the predicted effect sizes of GP73 on CAD were comparable and consistent in the direction across IVW, weighted median, and weighted mode method (all P-value<0.05; Table 2, Figure 2A). MR-Egger methods reported no horizontal pleiotropy (intercept=-0.006, se = 0.021, P=0.79; Table 2). Besides, the MR-PRESSO method did not detect outlying pQTL causing horizontal pleiotropy (P-value for MR-PRESSO global test = 0.19). In leave-one-out analysis, when singly withdrawing pQTLto assess the remaining effect, all estimates were consistent at each time, indicating that no pQTL substantially influenced overall estimation and the causal association was not biased by potential driving pQTL (Figure 2B). The scatter plot showed dose-response relationship between circulating GP73 level and the incidence of CAD (Figure 2C). Besides, no asymmetry was observed in the funnel plot, suggesting no horizontal pleiotropy of pQTLs (Figure S3).

When externally replicated in CAD GWAS of FINNGEN study, genetically determined GP73 level was still significantly associated with CAD risk; meanwhile, the causal estimates were comparable with that in the primary analysis (OR, 1.08 versus 1.11, Table S7). We further evaluated the causal relationship between genetically determined GP73 levels with other atherosclerosis diseases using the IVW method. As depicted in Table S8, genetically determined higher circulating GP73 concentration was associated with an increased risk of myocardial infarction (OR, 1.18; 95% CI, 1.09-1.28; P-value, <0.001), large artery atherosclerotic stroke (OR, 1.29; 95% CI, 1.07-1.55; P-value, 0.008) and PAD (OR, 1.001; 95% CI, 1.000-1.001; P-value, 0.02).

### Network Mendelian randomization

Network MR analysis showed that across metabolic risk factors profiles for CAD, GP73 level was causally associated with LDL-c and HbA1c, both of which were externally validated using other GWAS data (Table S9). Besides, LDL-c and HbA1c were causally associated with incident CAD (Table 3). Mediation analysis showed that they served as mediators in the causal pathway from GP73 to CAD and transmitted 42.1% and 8.7% of the total effects, respectively (Table 3).

### Vascular GP73 expression and atherosclerosis

**Figure 3:**
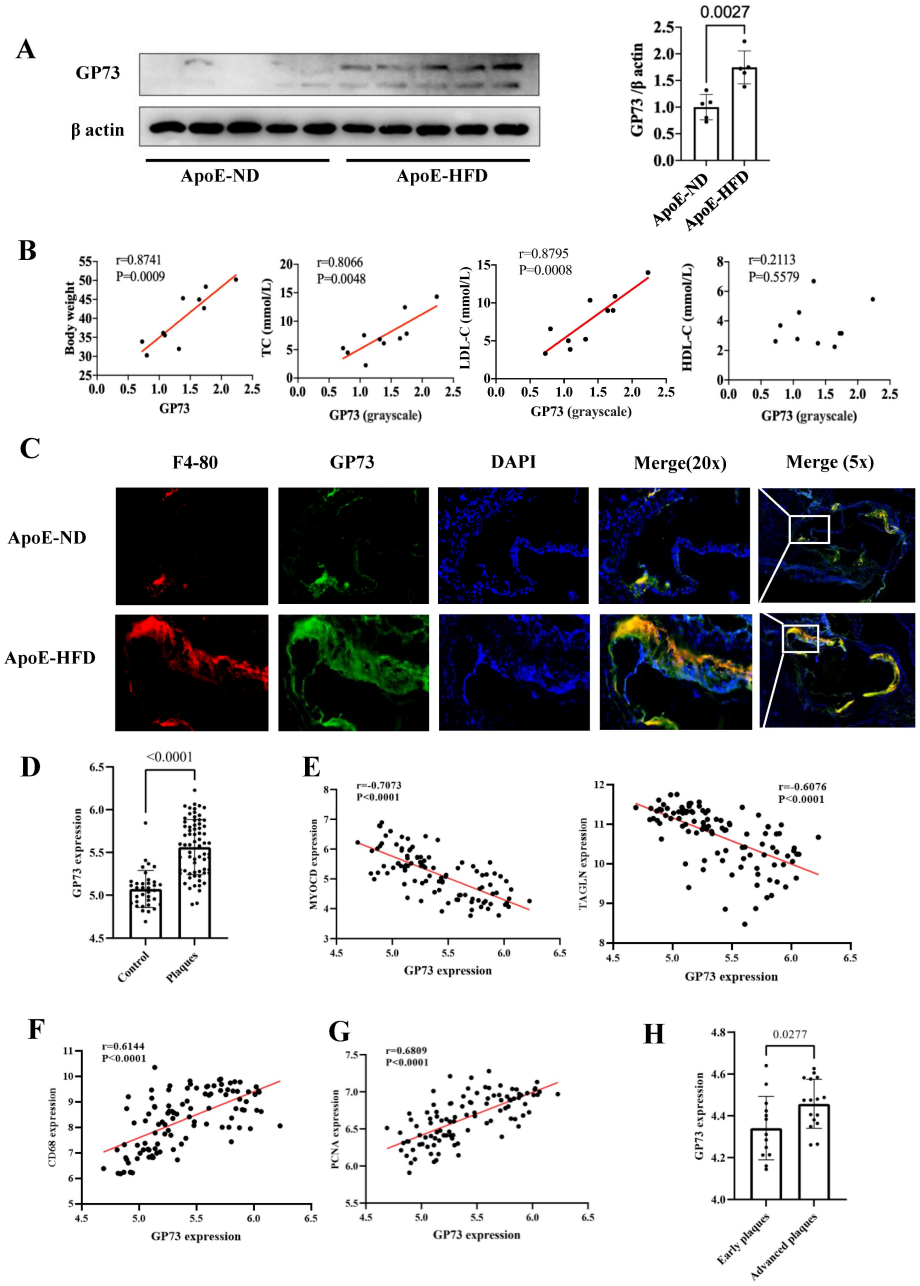
** GP73 is expressed in human and mouse atherosclerotic lesions. (A)** GP73 was upregulated in aortas from ApoE-HFD mice (n=5) than those from ApoE-ND mice (n=5). **(B)** Vascular GP73 expression was positively correlated with body weight, TC, and LDL-c in samples composed of 5 ApoE-HFD mice and 5 ApoE-ND mice. **(C)** Representative images showed GP73 (green) was co-localized with F4-80 (red) in the aortic section from ApoE-HFD mice, whilst fluorescence intensity of GP73 and F4-80 was markedly reduced in ApoE-ND mice. **(D and H)** GP73 mRNA expression was significantly upregulated in human peripheral arteries with atherosclerosis and advanced plaques in carotid arteries. **(E, F, and G)** In transcript levels, GP73 was negatively correlated with MYOCD and TAGLN, positively correlated with CD68 and PCNA. The samples were obtained from human peripheral arteries in GSE100927 **(D, E, F, G)** and human carotid arteries in GSE28829 **(H)** dataset. GP73, Golgi protein 73; ApoE-HFD, ApoE-/- mice with high-fat diet; ApoE-ND, ApoE-/- mice with normal diet; TC, total cholesterol; LDL-c, low-density lipoprotein cholesterol; MYOCD, myocardin; TAGLN, transgelin; PCNA, proliferating cell nuclear antigen.

Western blot and immunofluorescence staining demonstrated that GP73 expression in the aorta was significantly upregulated in ApoE-HFD mice compared with ApoE-ND mice (Figure 3A and C). Apparent colocalization of GP73 (green) and F4-80 (red) in the aortic section was observed in ApoE-HFD mice, while ApoE-ND mice had a considerably reduced colocalization area and diminished fluorescence intensity (Figure 3C). Besides, GP73 expression in the aorta was positively correlated with body weight, circulating TC, and LDL-c concentrations (Figure 3B). As for the human case, peripheral arteries with atherosclerotic plaques exhibited higher expression of GP73 than control arteries (Figure 3D). Using a sample composed of 69 atherosclerotic arteries and 35 control arteries, we observed reversal mRNA expression patterns of GP73 and contractile markers of vascular smooth muscle cells (VSMCs), including MYOCD (myocardin) and TAGLN (transgelin) (Figure 3E). On the opposite, we demonstrated strongly positive correlations in expression patterns between GP73 with proliferation marker of VSMCs (proliferating cell nuclear antigen, PCNA) and macrophage marker (CD68) (Fig 3F and G). Moreover, vascular GP73 expression became up-regulated with plaque progression, significantly higher in advanced plaque than early plaque (Figure 3H).

## Discussion

Using a strategy integrating protein arrays and causal inference, we first reported an association between higher genetically predicted circulating GP73 levels and increased CAD risk. Evidence from a network MR design showed that dyslipidemia and hyperglycemia transmitted the causal effects from GP73 to CAD. Apart from the circulating form of GP73, we also observed up-regulation of vascular GP73 expression upon atherosclerotic lesions, which may involve in macrophage recruitment and VSMCs phenotypic switching during atherosclerosis.

GP73, a type II Golgi transmembrane glycoprotein, is highly expressed in liver inflammation and a wide variety of tumors 43-45. Despite this, limited research has explored the role of GP73 in cardiovascular diseases. In our prior investigation, we demonstrated that GP73 promotes atherosclerosis by activating NF-κB/NLRP3 inflammasome signaling 46. Notably, the present study represents the first attempt to explore the epidemiological association between GP73 and CAD. Our results not only generate an effective biomarker for the identification of CAD but also provides a potential target for prevention and therapeutic intervention in CAD. The findings enrich the etiological information of CAD, facilitating further research to understand the pathophysiology underlying CAD. From the perspective of translational medicine, future fundamental research confirming and illustrating the underlying pathogenic mechanism of GP73 may help establish an effective strategy in the management of CAD. From an epidemiological perspective, considering the causal role of GP73 in the pathogenesis of CAD, a further prospective study focusing on the prognostic value of GP73 will provide evidence for clinical risk stratification of CAD in the general population. Besides, network MR analysis in the present study partially sheds light on the biological network from GP73 to CAD thus, targeted interventions acting on the identified mediators along the causal pathway, e.g., management of dyslipidemia and hyperglycemia, are also helpful to minimize the harmful effects of GP73 on CAD.

Contrary to the high burden of CAD, despite the technological advancement and substantial efforts in drug discovery for targeting CAD, new drug approval seems to stagnate recently 47. Summarized data from 218 failed drug trials showed that over half of the failures in phase Ⅱ and Ⅲ trials are attributed to the lack of efficacy 48. Pre-screening for causality of candidate drug target in disease etiology provides genetic evidence to anticipate the treatment efficacy before entry into clinical trial 47, 49. MR approach, analogous to a 'natural' randomized controlled trial, exhibited promising performance in predicting whether interventions on therapeutic target modifies the risk of disease 49. Being different from the emerging 'Phenome-wide Mendelian randomization' approach to systematically evaluate the causal effects of circulating proteome on diseases 50-52, we integrated the results from CAD biomarker study with causal inference, making our findings more reliable in the prediction of drug targets. Further research is warranted to validate and excavate the potential therapeutic value of GP73 on CAD.

Network MR in the present analysis provided insights into how GP73 induces CAD and highlighted that LDL-c serves as the most predominant mediator in the causal pathway from GP73 to CAD (Table 3). According to the GeneCards database (https://www.genecards.org) 53, regulation of lipid metabolic process is one of the biological processes of GOLM1, the gene coding for GP73 (Table S10). In line with our findings, Yang et al. elucidated that overexpression of GP73 in HepG2 and HL7702 cell enhanced SCAP-SREBPs binding, which in turn upregulated cholesterol synthesis-related gene expression and intracellular cholesterol level, leading to lipogenesis 54. On the other hand, here we found hyperglycemic condition is another potential intermediate in the atherogenic pathway downstream of GP73. Interestingly, Wan et al. found experimental administration of GP73 into mice led to immediate hyperglycemia and compensatory hyperinsulinemia, indicating that GP73 may serve as a “glucogenic hormone” 55, verifying our assumption from MR analysis. Despite all of these findings supporting the causal relationship between GP73 with LDL-c and HbA1c, further epidemiological surveys in population are still needed to verify whether GP73 induced glucose and lipid metabolism disorder, consequently causing CAD in the real world. If confirmed, intervention targeted on LDL-c and glucose may act as an alternative strategy to reduce the risk of CAD in individual genetic predisposition to high GP73 levels. On the contrary, GP73 may also provide new perspectives for managing dyslipidemia and hyperglycemia.

Both mouse models and human tissues confirmed higher vascular GP73 expression in the case of atherosclerosis, more pronounced in an advanced state; however, unlike the circulating form of GP73, there is currently no evidence to determine whether vascular GP73 up-regulation *in situ* is the result of atherosclerosis or serves as an initiator in the atherosclerotic process. Indeed, the correlations between GP73 and several atherosclerotic markers of plaques guide the direction for subsequent mechanistic studies. Macrophage infiltration into the artery is the key contributor in the atherosclerotic process, involving in plaque initiation, progression, and destabilization 56. Here, our findings revealed that macrophage recruitment was dramatically enhanced in the GP73 expression region in the atherosclerotic artery of ApoE-HFD mice. Additional research should elucidate whether inhibition of GP73 in arteries can attenuate macrophage recruitment and formation of foam cells, subsequently ameliorating atherosclerosis. Under physiologic circumstances, healthy VSMCs generate a series of contractile proteins to maintain their normal contractile function, e.g., MYOCD, TAGLN, and smooth muscle alpha actin 2 (ACTA2) 57, 58. During the atherosclerosis process, VSMCs decrease contractile gene expression in response to stimuli whilst switching to synthetic VSMCs, manifested as aberrant activation of proliferation and migration. Our study found vascular GP73 up-regulation *in situ* was concomitant with contractile phenotype loss and synthetic phenotype transition, which required silencing and overexpression experiments for functional validation.

### Strengths and limitations

Several methodological strengths should be mentioned in our study. Indeed, disparate lines of evidence, called “triangulation”, can produce a more convincing conclusion 59. The reliability of our research lies in the comprehensive evidence from patient-level data, causal inference, and mouse model. Additionally, sensitivity analyses verified the reliability and robustness of the results, including other optional MR methods, using other atherosclerosis outcomes sharing similar etiology and adequate external replications in MR analysis.

Despite the advantage, this study also has some limitations. First, the proteomics evidence was susceptible to the relatively small sample size and limited variety of protein measurements in the protein array. Even so, it still provided us with an alternative DEPs list from the clinical scenario, thus reinforcing the findings of causal inference. Second, considering the participants of GWAS used for our analysis were predominantly of European ancestry, it was unclear whether the evidence of causality could be generalizable to other racial groups. Nevertheless, the consistency of the findings between protein arrays using the Asian population (the RED-CARPET study) and causal inference from European ancestry alleviated this concern to some extent. Third, owing to the design of 2-sample MR, only summary-level estimates on genetic associations were used, therefore limiting some analyses requiring individual-level data, e.g., investigating the nonlinear causal effect of GP73, subgroup analyses to explore whether the association was modified by other factors. Fourth, only 6 among 18 candidate proteins acquired available genetic instruments from the AGES study. Further MR research incorporating more available GWAS on proteome may help to uncover additional causative agents of CAD. Last but not least, we observed different results in the causal association between GP73 and CAD risk using MR-Egger method and other MR methodology. Indeed, MR-Egger method is less precise in estimation of causal effects, especially with limited number of genetic instruments 26. Hence, MR-Egger method is mainly applied to test the pleiotropy instead of causal effect estimation 26.

## Conclusion

In conclusion, our study showed that genetic predisposition to higher circulating GP73 levels is associated with increased CAD risk, which was mainly mediated by dyslipidemia and hyperglycemia. Furthermore, upregulation of vascular GP73 expression is also correlated with the occurrence and progression of atherosclerosis. These findings suggest a potential direction for exploring unknown mechanisms in the pathogenesis of CAD, and subsequent in-depth research should focus on whether interventions targeting GP73 can ameliorate CAD risk.

## Supplementary Material

Supplementary figures, tables, methods.

## Figures and Tables

**Table 1:** Baseline characteristics of CAD group and control group

Characteristics	CAD group (n=33)	Control group (n=31)	P-value
Age, years	56.4±12.1	54.7±11.1	0.56
Men, %	21 (63.6)	19 (61.3)	0.85
Smoking, %	11 (33.3)	13 (41.9)	0.48
BMI, kg/m^2^	23.9±3.0	25.0±3.0	0.15
SBP, mmHg	127.6±19.7	125.0±15.0	0.56
DBP, mmHg	79.8±10.5	78.0±11.7	0.52
Hypertension, %	20 (60.6)	17 (54.8)	0.64
DM, %	9 (27.3)	8 (25.8)	0.89
TC, mmol/L	4.71±1.1	4.4±0.9	0.26
TG, mmol/L	2.1±1.4	1.7±0.8	0.17
LDL-c, mmol/L	3.0±0.7	2.7±0.7	0.12
HbA1c, %	6.6±2.1	6.0±0.9	0.16
CK-MB, ng/ml	3.2±3.3	1.3±0.6	0.003
cTNT, ng/ml	0.4±0.9	0.0±0.0	0.007
Gensini Score	88.2±52.4	0.0±0.0	<0.001

Data are presented as mean ± SD or number (percentage). Abbreviation: BMI, body mass index; SBP, systolic blood pressure; DBP, diastolic blood pressure; DM, diabetes mellitus; TC, total cholesterol; TG, triglycerides; LDL-C, low-density lipoprotein cholesterol; HDL-C, high-density lipoprotein cholesterol; HbA1c, glycated hemoglobin; CK-MB, Creatine kinase-MB; cTNT, Cardiac troponin T.

**Table 2:** Causal associations between genetically determined GP73 level and CAD

Exposure- outcome	Method	Causal estimate			
		pQTLs	OR	95% CI	P-value
GP73-CAD	Inverse variance weighted^a^	5	1.11	1.05-1.18	<0.001
	Weighted median	5	1.11	1.06-1.16	<0.001
	Weighted mode	5	1.11	1.06-1.17	0.02
	MR Egger	5	1.14	0.98-1.32	0.20
	MR-PRESSO	5	1.11	1.05-1.18	0.02
	Test for Heterogeneity: P=0.02 (MR-Egger) and P=0.05 (IVW)				
	Test for Horizontal pleiotropy: MR-Egger intercept=-0.006, se = 0.021, P=0.79				
	MR-PRESSO global test: P=0.19				

Abbreviations: GP73, golgi protein 73; CAD, coronary artery disease; OR, odds ratio; 95% CI, 95% confidential interval; FDR, false discovery rate. ^a^ Inverse variance weighted (random-effect) method

**Table 3:** Network Mendelian Randomization and mediation analysis between GP73 and CAD based on IVW method

Metabolic trait	Causal estimates between GP73 with traits (Discovery)				Causal estimates between traits with CAD				PM
	pQTLs	β	SE	P-value	pQTLs	OR	95%CI	P-value	
TC	3	0.072^ a^	0.056	0.20	-	-	-	-	-
HDL-c	3	0.032^ a^	0.048	0.51	-	-	-	-	-
LDL-c	3	0.099^ a^	0.049	0.04	40	1.58^ a^	1.43-1.74	<0.001	42.1%
HbA1c	5	0.033^ b^	0.007	<0.001	11	1.33**^ b^**	1.09-1.61	0.004	8.7%
HOMA-β	5	-0.002^ b^	0.007	0.78	-	-	-	-	-
HOMA-IR	5	0.011^ b^	0.008	0.18	-	-	-	-	-

Abbreviations: GP73, golgi protein 73; CAD, coronary artery disease; IVW, inverse variance weighted; CAD, coronary artery disease; TC, total cholesterol; LDL-c, low-density lipoprotein cholesterol; HDL-c, high-density lipoprotein cholesterol; HbA1c, glycated hemoglobin; HOMA-β, homeostasis model assessment of β-cell function; HOMA-IR, homeostatic model assessment for insulin resistance; PM, proportion mediated. ^a^ Inverse variance weighted (random-effect) method; ^b^ Inverse variance weighted (fixed-effect) method.
