# Supplementary material for: Causal Association of Golgi Protein 73 With Coronary Artery Disease: Evidence from Proteomics and Mendelian Randomization
Source: Int J Med Sci. 2024 Aug 12;21(11):2127–38. doi: 10.7150/ijms.94179 (PMC11373545; doi:10.7150/ijms.94179)
Supplement: Supplementary file 1 — Supplementary figures, tables, methods. [file ijmsv21p2127s1.pdf]

Causal Association of Golgi Protein 73 With Coronary Artery Disease: Evidence from Proteomics and Mendelian Randomization

Brief title: GP73 and CAD

Supplemental Material

Figure S1 Flowchart of study design

Figure S2 Framework of network Mendelian randomization

Figure S3 Funnel plot for MR analysis of causal effect of GP73 on CAD

Table S1 The genome-wide association studies (GWAS) in the Mendelian randomization study

Table S2 Genetic instruments in AGES study used in Mendelian randomization analysis

Table S3 The associations between genetic variant with outcome

Table S4 Description of GSE100927 and GSE28829

Table S5 Differentially expressed proteins between the CAD group and control group ($P\text{-value}<0.05$ and $\log_2FC>0.263$ or $\log_2FC< -0.263$)

Table S6 The causal association between candidate protein with CAD in Mendelian randomization

Table S7 Replication of causal association between GP73 level with coronary artery disease using CAD GWAS in FINNGEN study

Table S8 Causal associations between GP73 level and atherosclerosis disease

Table S9 Discovery and replication of causal association between GP73 and metabolic risk factors

Table S10 Gene Ontology (GO) - Biological Process for GOLM1 Gene

Text S1 Introduction on RED-CARPED study and data collection method

Text S2 Protein arrays protocol

Text S3 STROBE-MR: Guidelines for strengthening the reporting of Mendelian randomization studies

Text S4 Concept of mediation analysis

Text S5 Information on experimental procedures

Text S6 MEGASTROKE CONSORTIUM

Figure S1 Flowchart of study design

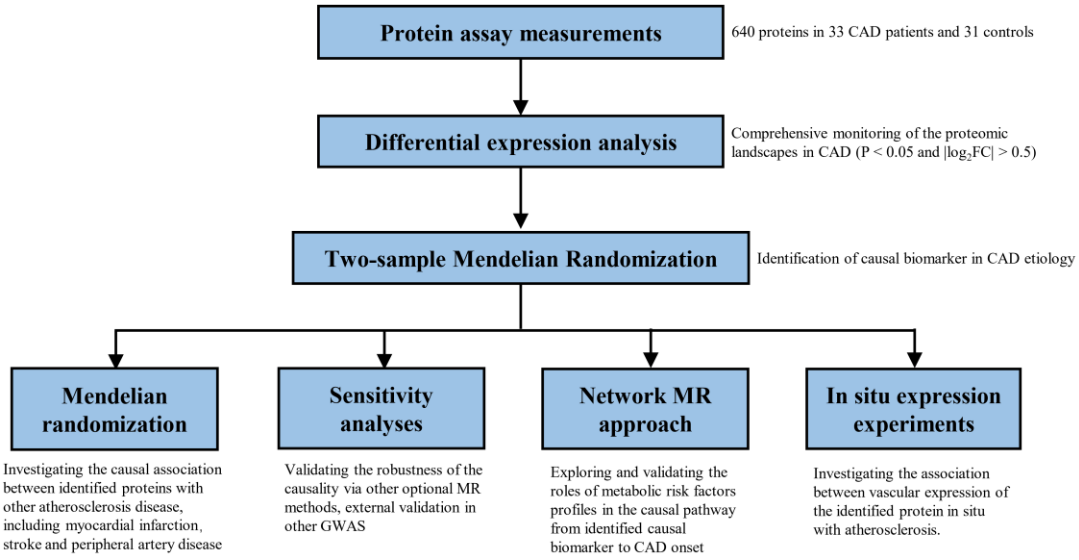

(1) Measurement of 640 circulating plasma proteins for via protein arrays in 33 coronary artery disease (CAD) patients and 31 controls; (2) Differential expression analysis was performed to panoramically identify proteins correlated with CAD; (3) Two-sample mendelian randomization was conducted to screen causative agents for CAD; (4) For the identified proteins, causal inferences on other atherosclerosis outcomes sharing similar etiology (myocardial infarction, stroke and PAD) were performed; (5) Further validating the robustness of the causality using other optional MR methods and external replication in other GWAS; (6) Performing network Mendelian randomization to explore the role of metabolic risk factors of CAD in the causal pathway from identified causal agents to CAD; (7) Investigating the association between vascular expression of the identified protein in situ with atherosclerosis using both mouse and human samples.

Figure S2 Framework of network Mendelian randomization[1]

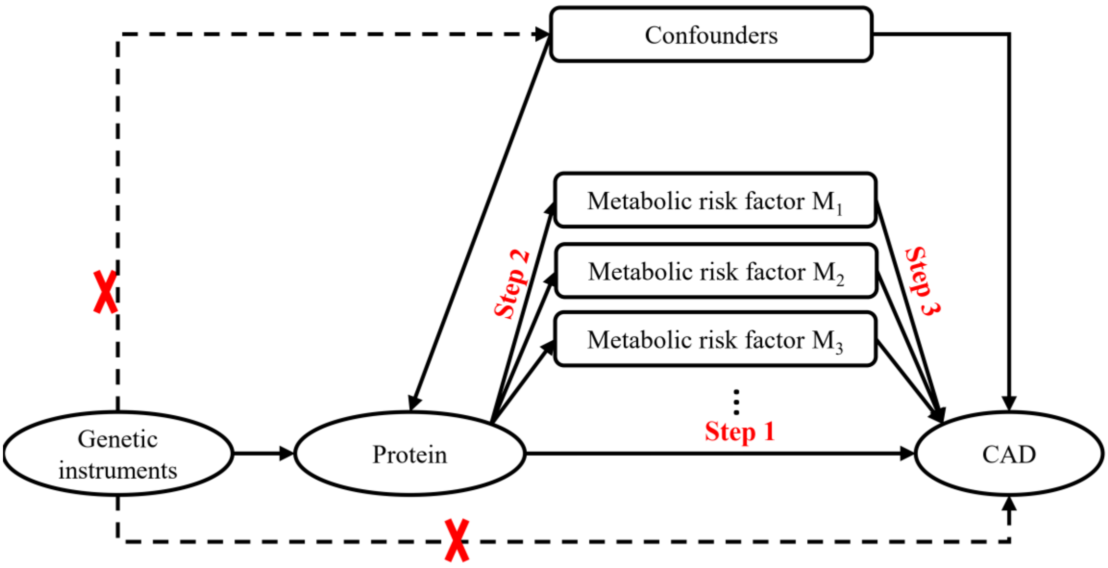

Step 1: Assess the causality between candidate protein with CAD; Step 2: Assess the causality between candidate protein with metabolic risk factors; Step 3: Assess the causality between metabolic risk factors with CAD. Only when causality exists in all three steps, the metabolic risk factor was considered as a mediator.

Figure S3 Funnel plot for MR analysis of causal effect of GP73 on CAD

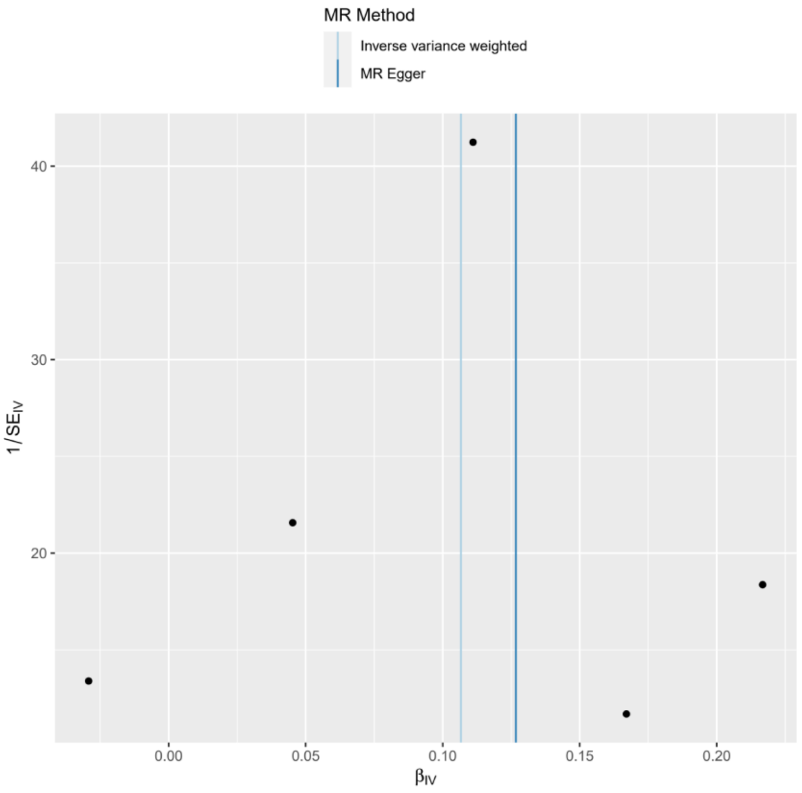

Table S1 The genome-wide association studies (GWAS) in the Mendelian randomization study

Outcome	GWAS	Phenotypes	Ancestry	Adjustments	pQTL	Sample size	URL for data download
CAD (Discovery)	CARDIoGRAMplus C4D 1000 Genomes-based GWAS	MI, ACS, chronic stable angina, or coronary stenosis of >50%	Multi-ancestry (77% European)	Age, gender	9,455,779	60,801 cases /123,504 controls	http://www.cardiogramplusc4d. org/data-downloads/[2]
CAD (Replication)	FinnGen study	Angina pectoris, MI, IHD, cardiac arrest, and other unattended or cause unknown sudden death	European	Age, gender	16,380,466	21,012 cases / 197,780 controls	http://www.finngen.fi/fi
Stroke	MEGASTROKE	AIS and subtypes (LAS, SVS)	Multi-ancestry	Age, gender	~8,000,000	67,162 cases /454,45 controls	http://megastroke.org/download.html[3]
PAD	UK Biobank	PAD	European	Age, gender	9,637,467	1,230 cases /359,964 controls	http://www.nealelab.is/ukbiobank
Lipid profile (Discovery)	GLGC	LDL-c, HDL-c, TC	Multi-ancestry	Age, gender	2,447,422	188,577	http://lipidgenetics.org/[4]
Lipid profile (Replication)	BioBank Japan	LDL-c	East Asian	Age, gender	6,108,953	72,866	http://jenger.riken.jp/en/ [5]
Glycemic profile (Discovery)	MAGIC	HbA1c，HOMA-β HOMA-IR	European	Age, gender	~2,500,000	46,368	https://magicinvestigators.org/[6-7]
Glycemic profile (Replication)	UK Biobank	HbA1c	European	Age, gender	13,586,180	344,182	http://www.nealelab.is/ukbiobank

Abbreviation:

Outcome name: CAD, coronary artery disease; PAD, peripheral artery disease.

GWAS names: CARDIoGRAMplusC4D, Coronary ARtery DIsease Genome-wide Replication and Meta-analysis (CARDIoGRAM) plus The Coronary Artery Disease (C4D) Genetics; GLGC, Global Lipids Genetics Consortium; MAGIC, Meta-Analyses of Glucose and Insulin-related traits Consortium.

Phenotype names: MI, myocardial infarction; ACS, acute coronary syndrome; IHD, ischemic heart disease; AIS, any ischemic stroke; LAS, large artery stroke; SVS, small vessel stroke; PAD, peripheral artery disease; LDL-c, low-density lipoprotein cholesterol; HDL-c, high-density lipoprotein cholesterol; TC, total cholesterol; HbA1c, glycated hemoglobin; HOMA-β, homeostasis model assessment of β-cell function; HOMA-IR, homeostatic model assessment for insulin resistance.

Table S2 Genetic instruments in AGES study used in Mendelian randomization analysis

Uniprot ID	Protein name	pQTL	CHR: POS	Sample size	EA	EAF	β	SE	P-value	F-statistics
P02741	C-reactive protein	rs876537	1:31613465	5343	T	0.3712	-0.153	0.01999	2.29E-14	58.58
		rs7953249	12:31612724	5342	G	0.3915	-0.126	0.01952	1.17E-10	41.67
		rs2075650	19:45395619	5343	G	0.153	-0.1848	0.02663	4.45E-12	48.16
P05362	Intercellular adhesion molecule 1	rs657152	9:136139265	5340	T	0.279	-0.156	0.02121	2.19E-13	54.10
		rs3745600	19:10224526	5333	T	0.474	-0.1264	0.01897	2.99E-11	44.40
		rs34495400	19:10664661	5341	T	0.008878	0.6987	0.1012	5.75E-12	47.67
		rs55762744	19:10488926	5341	T	0.005504	0.8845	0.1275	4.52E-12	48.13
		rs8109578	19:31613048	5335	A	0.09154	-0.2322	0.03232	7.61E-13	51.62
		rs113197610	19:10363251	5341	C	0.02486	-0.4727	0.06119	1.33E-14	59.68
		rs5030400	19:10395796	5341	T	0.009677	0.9559	0.09907	7.53E-22	93.10
		rs34536443	19:10463118	5341	C	0.04252	0.4857	0.04665	3.79E-25	108.40
		rs2304256	19:10475652	5341	A	0.2579	0.3423	0.02119	2.31E-57	260.95
		rs2230399	19:10444603	5341	G	0.0799	0.6239	0.03426	6.30E-72	331.63
		rs1799969	19:10394792	5341	A	0.1349	-0.7812	0.02589	8.807E-185	910.46
		rs2228615	19:10403368	5340	A	0.3974	-1.014	0.01358	0	5575.40
P09341	Growth-regulated alpha protein	rs2251746	1:159272060	5335	C	0.2805	0.1396	0.02011	4.33E-12	48.19
		rs12075	1:159175354	5334	G	0.4357	-0.1978	0.01791	4.77E-28	121.97
		rs241771	17:26592946	5334	T	0.4498	-0.1831	0.01795	3.18E-24	104.05
P12821	Angiotensin-converting enzyme	rs9898	3:31615480	5335	T	0.3004	0.1868	0.02057	1.52E-19	82.47

		rs4962153	9:136323754	5336	A	0.09641	-0.2202	0.03191	5.75E-12	47.62
		rs4968639	17:61055390	5335	G	0.2149	-0.2279	0.02284	2.95E-23	99.56
		rs3730025	17:61557773	5336	G	0.01696	-1.181	0.07238	1.79E-58	266.23
		rs4343	17:61566031	5336	A	0.4999	-0.5652	0.01733	6.51E-213	1063.67
P14210	Hepatocyte growth factor	rs2511241	11:72945341	5340	C	0.08638	-0.3751	0.03387	3.39E-28	122.65
P15941	Mucin-1	rs241771	17:26592946	5327	T	0.4498	-0.1147	0.01748	5.92E-11	43.06
		rs9910163	17:31615623	5333	A	0.2539	-0.2035	0.02109	7.51E-22	93.11
P22894	Neutrophil collagenase	rs3130481	6:31839756	5339	G	0.4788	-0.12	0.01879	1.84E-10	40.79
		rs2857105	6:32790059	5340	G	0.02779	0.3615	0.0565	1.72E-10	40.94
		rs6924270	6:30681980	5339	G	0.01092	0.5813	0.09072	1.60E-10	41.06
		rs154989	6:32877173	5340	T	0.4611	0.1223	0.01905	1.48E-10	41.22
		rs11963870	6:30735229	5340	A	0.01589	0.4782	0.0743	1.33E-10	41.42
		rs2523674	6:31436789	5339	C	0.4007	0.123	0.01896	9.45E-11	42.09
		rs1705003	6:33385953	5338	G	0.1083	0.199	0.03065	9.27E-11	42.15
		rs2075798	6:31846741	5340	A	0.08034	0.2274	0.03421	3.29E-11	44.18
		rs138097363	6:32942386	5340	A	0.003285	1.084	0.1624	2.72E-11	44.55
		rs141444056	6:33283955	5340	T	0.006392	0.7902	0.1167	1.43E-11	45.85
		rs6457452	6:31795550	5338	T	0.0444	0.311	0.04593	1.42E-11	45.85
		rs61751507	10:101829514	5338	T	0.0453	-0.3181	0.04455	1.05E-12	50.98
		rs2846365	11:102571892	5342	A	0.4653	-0.1505	0.01931	7.65E-15	60.74
		rs2511241	11:72945341	5343	C	0.08638	-0.3502	0.03446	4.91E-24	103.28
		rs35231465	11:102584135	5343	A	0.02193	-0.9681	0.06474	1.48E-49	223.61
P48023	Tumor necrosis factor ligand	rs7617480	3:31611817	5339	A	0.2055	-0.152	0.02363	1.36E-10	41.38

	superfamily member 6	rs6762477	3:50093209	5339	G	0.4336	-0.1251	0.01921	8.13E-11	42.41
		rs6442117	3:48419897	5340	C	0.4678	0.1303	0.01911	1.01E-11	46.49
		rs146179438	3:48229366	5341	A	0.01341	0.5601	0.08117	5.80E-12	47.61
		rs17080138	3:48414274	5341	T	0.0237	0.4416	0.0632	3.15E-12	48.82
		rs388483	3:50615539	5340	G	0.4638	-0.1341	0.01903	2.11E-12	49.66
		rs35761247	3:48623124	5337	A	0.06503	0.314	0.03856	4.74E-16	66.31
		rs739983	3:50178011	5341	G	0.3127	0.1809	0.02056	1.82E-18	77.42
		rs9834639	3:31615434	5341	T	0.06294	0.4298	0.03949	2.63E-27	118.46
		rs2005557	3:49701298	5340	G	0.4782	0.2487	0.01867	7.14E-40	177.44
P49862	Kallikrein-7	rs61729512	12:7637769	5325	A	0.1165	0.1931	0.02908	3.48E-11	44.09
		rs1654528	19:51480840	5324	G	0.04049	-0.3927	0.0467	5.24E-17	70.71
P50895	Basal Cell Adhesion Molecule	rs505922	9:136149229	5332	C	0.2571	0.1353	0.02085	9.47E-11	42.11
		rs7853989	9:31612595	5331	C	0.09005	0.2315	0.03166	2.98E-13	53.47
P55287	Cadherin-11	rs4967857	16:64886822	5335	C	0.4087	-0.1298	0.0189	7.19E-12	47.17
		rs141063325	16:65005934	5337	A	0.009499	-0.7798	0.09652	8.05E-16	65.27
		rs35195	16:65025718	5335	A	0.3409	0.1616	0.01967	2.69E-16	67.50
		rs9910163	17:31615725	5317	A	0.2539	-0.2178	0.01948	1.10E-28	125.01
		rs241771	17:26592946	5316	T	0.4498	0.2216	0.01702	3.75E-38	169.52
P78380	Oxidized low-density lipoprotein receptor 1	rs2511241	11:72945341	5343	C	0.08638	-0.2886	0.03455	8.44E-17	69.77
Q76LX8	A disintegrin and metalloproteinase with thrombospondin motifs 13	rs142572218	9:136319670	5334	T	0.002663	-1.49	0.1814	2.68E-16	67.47
		rs1053878	9:136131651	5333	A	0.06109	0.335	0.03975	4.50E-17	71.03
		rs8176720	9:31613094	5334	C	0.3888	-0.1805	0.01949	2.90E-20	85.77

		rs34024143	9:136287582	5333	T	0.1465	-0.3216	0.0264	1.10E-33	148.40
		rs28515121	9:136291594	5332	A	0.4715	-0.3227	0.01866	3.01E-65	299.07
Q8NBJ4	Golgi membrane protein 1/ Golgi protein 73	rs549446	9:136135238	5336	T	0.2986	-0.1306	0.0203	1.36E-10	41.39
		rs4962153	9:136323754	5336	A	0.09641	0.2784	0.03092	2.97E-19	81.07
		rs1053878	9:136131651	5335	A	0.06109	0.3749	0.03843	2.68E-22	95.17
		rs505922	9:136149229	5335	C	0.2571	0.3908	0.02045	9.05E-79	365.19
		rs2287921	19:49228272	5336	T	0.3936	0.134	0.0188	1.16E-12	50.80
Q92563	Testican-2	rs148641213	10:73827379	5336	A	0.008347	-0.7373	0.1054	3.02E-12	48.93
		rs1245541	10:73849639	5335	T	0.4131	0.1565	0.01935	7.53E-16	65.41
Q9BY76	Angiopoietin-related protein 4	rs9910163	17:31615950	5327	A	0.2539	0.1478	0.02057	7.64E-13	51.63
		rs241771	17:26592946	5326	T	0.4498	-0.1779	0.01797	6.79E-23	98.01
Q9UNG2	Tumor necrosis factor ligand superfamily member 18	rs9910163	17:31616047	5314	A	0.2539	-0.1256	0.01871	2.09E-11	45.06

bbreviation: CHR: chromosome; POS: position; EA: effect allele; EAF: effect allele frequency.

Table S3 The associations between genetic variant with outcome

pQTL	Gene	EA	Association parameters for CAD (CARDIoGRAMplusC4D)		Association parameters for CAD in FINNGEN		Association parameters for MI		Association parameters for any ischemic stroke		Association parameters for large artery stroke	
			effect	se	effect	se	effect	se	effect	se	effect	se
rs1053878	*ABO*	A	0.01696	0.01738	-0.00720	0.02100	0.01108	0.02007	-0.00260	0.01950	0.06810	0.05470
rs2287921	*RASIP1*	T	-0.00392	0.01001	-0.00360	0.01300	-0.00177	0.01101	-0.01950	0.01000	0.47860	-0.04170
rs4962153	*ADAMTS13*	A	0.06034	0.01516	0.04340	0.01810	0.07396	0.01688	0.03760	0.01740	0.14980	0.07810
rs505922	*ABO*	C	0.04341	0.00948	0.03470	0.01300	0.07653	0.01058	-0.04980	0.01160	0.65520	-0.13710
rs549446	*ABO*	T	-0.02182	0.01117	-0.02580	0.01690	-0.02921	0.01249	-0.01330	0.01330	0.29900	-0.04610

pQTL	Gene	EA	Association parameters for small vessel stroke		Association parameters for peripheral artery disease		Association parameters for LDL-c (GLGC)		Association parameters for HDL-c		Association parameters for TC	
			effect	se	effect	se	effect	se	effect	se	effect	se
rs1053878	*ABO*	A	-0.01500	0.04740	0.00018	0.00027	0.00350	0.01160	-0.01620	0.01050	-0.01170	0.01070
rs2287921	*RASIP1*	T	-0.03330	0.02290	-0.00014	0.00014						
rs4962153	*ADAMTS13*	A	0.03860	0.03650	0.00024	0.00020	0.04570	0.00730	0.01380	0.00690	0.03430	0.00700
rs505922	*ABO*	C	0.00500	0.02640	0.00033	0.00015						
rs549446	*ABO*	T	0.05270	0.02940	-0.00009	0.00016	-0.01260	0.00600	-0.02070	0.00570	-0.02080	0.00570

pQTL	Gene	EA	Association parameters for LDL-c (Biobank Japan)		Association parameters for HbA1c (MAGIC)		Association parameters for HOMA-β		Association parameters for HOMA-IR		Association parameters for HbA1c (UK Biobank)	
			effect	se	effect	se	effect	se	effect	se	effect	se

rs1053878	*ABO*	A	0.04679	0.00626	0.01250	0.00690	-0.00180	0.00630	-0.00130	0.00800	0.08154	0.03006
rs2287921	*RASIP1*	T	-0.01037	0.01177	0.00390	0.00360	0.00360	0.00340	0.00260	0.00410	0.02804	0.01542
rs4962153	*ADAMTS13*	A	-0.02085	0.00847	0.01660	0.00540	-0.00040	0.00560	0.00740	0.00670	0.17399	0.02246
rs505922	*ABO*	C	0.02504	0.00523	0.01020	0.00350	-0.00170	0.00340	0.00330	0.00420	0.16345	0.01652
rs549446	*ABO*	T	-0.01480	0.00591	-0.00640	0.00400	0.00050	0.00400	-0.00480	0.00480	-0.06023	0.01782

Table S4 Description of GSE100927 and GSE28829

**Accession number**	**GSE100927**[8]	**GSE28829**[9]
Title	Atherosclerotic and control peripheral arteries gene expression	Gene Expression in early and advanced atherosclerotic plaque from human carotid arteries
Organism	Homo sapiens	Homo sapiens
Experiment type	Expression profiling by array	Expression profiling by array
Summary	Transcriptome analysis of human peripheral arteries from carotid, femoral and infra-popliteal territories in atherosclerotic and control tissue	To identify genes and pathways involved in the progression of atherosclerotic plaques from early to advanced stage in humans
Overall design	Atherosclerotic lesions (n=69) and control arteries (n=35) without atherosclerotic lesions (from deceased organ donors) were obtained from carotid, femoral and infra-popliteal arteries	Samples were extracted from atherosclerotic carotid artery segments, from early ((pathological) intimal thickening and intimal xanthoma, n=13) and from advanced (thin or thick fibrous cap atheroma, n=16) lesions

Table S5 Differentially expressed proteins between the CAD group and control group (P-value<0.05 and $log_2FC>0.263$ or $log_2FC< -0.263$)

Protein name	Uniprot ID	Fold change (CAD /Control)	P-value	Direction
IL-17B	Q9UHF5	-0.43502	0.000791	Down-regulated
ACE	P12821	-0.60947	0.002798	Down-regulated
FAS L	P48023	-0.31654	0.003452	Down-regulated
LOX-1	P78380	0.60751	0.004407	Up-regulated
Periostin	Q15063	0.947869	0.005337	Up-regulated
MMP-8	P22894	0.663479	0.007132	Up-regulated
Kallikrein 7	P49862	-0.37082	0.009262	Down-regulated
Thrombospondin-5	P49747	1.175349	0.018151	Up-regulated
Testican 2	Q92563	0.290883	0.015549	Up-regulated
Midkine	P21741	0.448534	0.018224	Up-regulated
ICAM-1	P05362	-0.29314	0.029055	Down-regulated
ANGPTL4	Q9BY76	-0.54942	0.018489	Down-regulated
ADAMTS13	Q76LX8	-0.40764	0.018121	Down-regulated
FSH	P01225	-0.76342	0.019319	Down-regulated
HTRA2	O43464	0.302938	0.019246	Up-regulated
Cadherin-11	P55287	-0.54401	0.018224	Down-regulated
Hepsin	P05981	0.302118	0.019696	Up-regulated
CA15-3	P15941	-0.65434	0.021515	Down-regulated
MCP-4	Q99616	0.354489	0.022022	Up-regulated
HGF	P14210	0.352365	0.02396	Up-regulated
Adipsin	P00746	-0.90153	0.0244	Down-regulated
GP73	Q8NBJ4	0.361246	0.033029	Up-regulated
IL-3	P08700	-0.55323	0.031375	Down-regulated
CRP	P02741	-0.72296	0.027308	Down-regulated
Renin	P00797	0.516599	0.034687	Up-regulated
EMMPRIN	P35613	0.541476	0.039866	Up-regulated
FLRG	O95633	0.578827	0.039792	Up-regulated
BCAM	P50895	-0.50288	0.041368	Down-regulated
Endoglycan	Q9NZ53	-0.32526	0.047729	Down-regulated
GITR L	Q9UNG2	0.48922	0.041055	Up-regulated
GROa	P09341	-0.38825	0.048254	Down-regulated
AFP	P02771	-0.40215	0.047859	Down-regulated

Table S6 The causal association between candidate protein with CAD in Mendelian randomization

UniprotID	Gene symbol	Protein full name	$P_{heterogeneity}$	No. of Instruments	OR	95%CI	P-value	FDR
Q8NBJ4[a]	*GOLM1*	Golgi membrane protein 1, Golgi protein 73	0.05	5	1.11	1.05-1.18	<0.001	0.005
P48023[a]	*FASLG*	Tumor necrosis factor ligand superfamily member 6	0.01	10	0.93	0.88-0.99	0.02	0.15
Q76LX8[b]	*ADAMTS13*	A disintegrin and metalloproteinase with thrombospondin motifs 13	0.11	4	1.04	1.00-1.09	0.05	0.23
P02741[a]	*CRP*	C-reactive protein	<0.001	3	0.82	0.66-1.01	0.07	0.23
P05362[a]	*ICAM1*	Intercellular adhesion molecule 1	<0.001	11	0.98	0.95-1.00	0.07	0.23
Q9BY76[b]	*ANGPTL4*	Angiopoietin-related protein 4	0.26	2	0.93	0.86-1.02	0.11	0.31
P78380[c]	*OLR1*	Oxidized low-density lipoprotein receptor 1	-	1	0.91	0.78-1.07	0.25	0.48
P15941[b]	*MUC1*	Mucin-1	0.12	2	0.95	0.87-1.04	0.25	0.48
P14210[c]	*HGF*	Hepatocyte growth factor	-	1	0.93	0.82-1.05	0.25	0.48
P55287[b]	*CDH11*	Cadherin-11	0.29	4	1.03	0.97-1.08	0.34	0.57
P49862[b]	*KLK7*	Kallikrein-7	0.56	2	0.97	0.88-1.07	0.57	0.88
P50895[a]	*BCAM*	Basal Cell Adhesion Molecule	<0.001	2	1.09	0.75-1.57	0.67	0.94
P09341[b]	*CXCL1*	Growth-regulated alpha protein	0.08	3	0.99	0.93-1.05	0.74	0.94

Q92563[c]	*SPOCK2*	Testican-2	-	1	0.99	0.88-1.12	0.88	0.94
P22894[b]	*MMP8*	Neutrophil collagenase	0.40	7	1.00	0.95-1.04	0.89	0.94
P12821[a]	*ACE*	Angiotensin-converting enzyme	<0.001	5	1.00	0.95-1.06	0.89	0.94
Q9UNG2[c]	*TNFSF18*	Tumor necrosis factor ligand superfamily member 18	-	1	1.00	0.83-1.19	0.97	0.97

Abbreviations: CAD, coronary artery disease; OR, odds ratio; 95% CI, 95% confidential interval; FDR, false discovery rate.

[a] Inverse variance weighted (random-effect) method;

[b] Inverse variance weighted (fixed-effect) method;

[c] Wald ratio method

Table S7 Replication of causal association between GP73 level with coronary artery disease using CAD GWAS in FINNGEN study

Exposure-outcome	Method	Causal estimate			
		pQTL	OR	95% CI	P-value
GP73-CAD	Inverse variance weighted[a]	5	1.08	1.03-1.13	0.003
	Weighted median	5	1.08	1.02-1.14	0.01
	Weighted mode	5	1.09	1.02-1.17	0.06
	MR Egger	5	1.07	0.91-1.28	0.47
	Test for Heterogeneity: P=0.09 (MR-Egger) and P=0.16 (IVW)				
	Test for Horizontal pleiotropy: MR-Egger intercept=0.001, se = 0.025, P=0.98				

[a] Inverse variance weighted (fixed-effect) method

Table S8 Causal associations between GP73 level and atherosclerosis disease

Secondary outcome	Method	Causal estimate			
		pQTL	OR	95% CI	P-value
Myocardial infarction	Inverse variance weighted [a]	5	1.18	1.09-1.28	<0.001
	Weighted median	5	1.19	1.13-1.26	<0.001
	Weighted mode	5	1.23	1.15-1.30	0.003
	MR Egger	5	1.23	1.00-1.52	0.14
	Test for Heterogeneity: P=0.002 (MR-Egger) and P=0.004 (IVW)				
	Test for Horizontal pleiotropy: MR-Egger intercept=-0.014, se = 0.03, P=0.68				
Ischemic stroke	Inverse variance weighted [a]	5	1.08	0.99-1.18	0.07
	Weighted median	5	1.13	1.07-1.19	<0.001
	Weighted mode	5	1.14	1.07-1.21	0.01
	MR Egger	5	1.20	1.00-1.43	0.14
	Test for Heterogeneity: P=0.02 (MR-Egger) and P=0.004 (IVW)				
	Test for Horizontal pleiotropy: MR-Egger intercept=-0.031, se = 0.024, P=0.29				
Large artery atherosclerotic stroke	Inverse variance weighted [a]	5	1.29	1.07-1.55	0.008
	Weighted median	5	1.37	1.21-1.56	<0.001
	Weighted mode	5	1.41	1.22-1.63	0.01
	MR Egger	5	1.67	1.17-2.38	0.07
	Test for Heterogeneity: P=0.09 (MR-Egger) and P=0.02 (IVW)				
	Test for Horizontal pleiotropy: MR-Egger intercept=-0.078, se = 0.048, P=0.20				
Small vessel stroke	Inverse variance weighted [b]	5	0.97	0.87-1.07	0.49
	Weighted median	5	0.98	0.87-1.10	0.76
	Weighted mode	5	0.99	0.87-1.13	0.92
	MR Egger	5	1.17	0.94-1.46	0.26
	Test for Heterogeneity: P=0.46 (MR-Egger) and P=0.19 (IVW)				
	Test for Horizontal pleiotropy: MR-Egger intercept=-0.056, se = 0.030, P=0.16				
Peripheral artery disease	Inverse variance weighted [b]	5	1.001	1.000-1.001	0.02
	Weighted median	5	1.001	1.000-1.001	0.02
	Weighted mode	5	1.001	1.000-1.002	0.07
	MR Egger	5	1.001	1.000-1.003	0.13
	Test for Heterogeneity: P=0.67 (MR-Egger) and P=0.55 (IVW)				
	Test for Horizontal pleiotropy: MR-Egger intercept<0.001, se <0.001, P=0.31				

[a] Inverse variance weighted (random-effect) method;

[b] Inverse variance weighted (fixed-effect) method.

Table S9 Discovery and replication of causal association between GP73 and metabolic risk factors

Metabolic trait	Causal estimates between GP73 with traits (Discovery)				Causal estimates between GP73 with traits (Replication)			
	**pQTL**	**β**	**SE**	**P-value**	**pQTL**	**β**	**SE**	**P-value**
TC	3	0.072 [a]	0.056	0.20	-	-	-	-
HDL-c	3	0.032 [a]	0.048	0.51	-	-	-	-
LDL-c	**3**	**0.099** [a]	**0.049**	**0.04**	**5**	**0.071** [a]	**0.029**	**0.02**
HbA1c	**5**	**0.033** [b]	**0.007**	**<0.001**	**5**	**0.405** [a]	**0.063**	**<0.001**
HOMA-β	5	-0.002 [b]	0.007	0.78	-	-	-	-
HOMA-IR	5	0.011 [b]	0.008	0.18	-	-	-	-

Abbreviation: IVW, inverse variance weighted; CAD, coronary artery disease; TC, total cholesterol; LDL-c, low-density lipoprotein cholesterol; HDL-c, high-density lipoprotein cholesterol; HbA1c, glycated hemoglobin; HOMA-β, homeostasis model assessment of β-cell function; HOMA-IR, homeostatic model assessment for insulin resistance.

[a] Inverse variance weighted (random-effect) method;

[b] Inverse variance weighted (fixed -effect) method.

Table S10 Gene Ontology (GO) - Biological Process for GOLM1 Gene

GO ID	Qualified GO term	Evidence
GO:0006997	nucleus organization	IEA [a]
GO:0019216	regulation of lipid metabolic process	IEA [a]
GO:0043687	post-translational protein modification	TAS [b]
GO:0044267	cellular protein metabolic process	TAS [b]

[a] IEA: Inferred from Electronic Annotation;

[b] TAS, Traceable Author Statement.

Text S1 Introduction on RED-CARPED study and data collection method

REal-world Data of CARdiometabolic ProtEcTion is a single-center, ambispective cohort study aimed at identifying risk factors associated with metabolic cardiovascular diseases and explore their relationship with long-term cardiovascular endpoints (registration number: ChiCTR2000039901). This is achieved through conducting long-term follow-up of patients with metabolic cardiovascular diseases in a real-world setting. Patients admitted to the Cardiology department of the First Affiliated Hospital of Sun Yat-sen University with metabolic cardiovascular diseases between 2003 and 2033 were consecutively enrolled. The registry includes patients with any of the following conditions: coronary heart disease, hypertension, heart failure, stroke, diabetes, obesity, dyslipidemia, or hyperuricemia. From 2017 to 2018, a total of 2,361 patients (67.6% male; mean age 63.5±11.7 years) were diagnosed with coronary heart disease in the RED-CARPET study, among whom 64 patients were randomly selected for high-throughput proteomics.

Information on gender, age, and smoking status were self-reported. After a 15 minutes rest in a sitting position, systolic blood pressures (SBPs) and diastolic blood pressures (DBPs) were measured three times every 5 minutes at right arm using mercury sphygmomanometer, and the mean values were used for analysis. Standing height and weight were measured with the subjects wearing light clothes without shoes in a standardized posture. Body mass index (BMI) was calculated as weight (in kilograms) divided by squared height (in meters). Hypertension (HTN) was ascertained if a participant has systolic blood pressure ≥ 140 mmHg or diastolic blood pressure ≥ 90 mmHg, prior history of hypertension, or antihypertensive medication[10]. We defined diabetes mellitus (DM) as fasting glucose ≥126 mg/dL, non-fasting glucose ≥ 200 mg/dL, A1C ≥ 6.5%, self-report of a previous diabetes diagnosis, or taking medication for diabetes[11]. Total cholesterol, low-density lipoprotein cholesterol, and triglycerides were detected by the automatic enzyme method. Glycated hemoglobin (HbA1c) was tested via automated immunochemistry method. Creatine kinase-MB (CK-MB), and Cardiac troponin T (cTNT) were measured using immunoassays.

Text S2 Protein arrays protocol

A. Completely Air Dry The Glass Slide

1. Take out the glass slide from the box, and let it equilibrate to room temperature inside the sealed plastic bag for 20-30 minutes. Remove slide from the plastic bag, peel off the cover film, and let it air dry for another 1-2 hours.

Incomplete drying of slides before use may cause the formation of “comet tails,” thin directional smearing of antibody spots.

B. Blocking & Incubation

2. Add 100 µl Sample Diluent into each well and incubate at room temperature for 30 minutes to block slides.

3. Decant buffer from each well. Add 100 µl of sample to each well. Incubate arrays at room temperature for 1-2 hour.

Longer incubation time is preferable for higher signals. This step may be done overnight at 4°C.

We recommend using 50 to 100 µl of original or diluted serum, plasma, conditioned media, or other body fluid, or 50-500 µg/ml of protein for cell and tissue lysates. Cover the incubation chamber with adhesive film during incubation, especially if less than 70 ul of sample or reagent is used.

4. Wash:

- Decant the samples from each well, and wash 5 times (5 min each) with 150 µl of 1X Wash Buffer I at room temperature with gentle shaking. Completely remove wash buffer in each wash step. Dilute 20x Wash Buffer I with H2O.
- (Optional for Cell and Tissue Lysates) Put the glass slide with frame into a box with 1X Wash Buffer I (cover the whole glass slide and frame with Wash Buffer I), and wash at room temperature with gentle shaking for 20 min.
- Decant the 1x Wash Buffer I from each well, wash 2 times (5 min each) with 150 µl of 1X Wash Buffer II at room temperature with gentle shaking.

  Completely remove wash buffer in each wash step. Dilute 20X Wash Buffer II with H2O.

“Incomplete removal of the wash buffer in each wash step may cause “dark spots,” the background signals higher than the spots.

C. Incubation with Biotinylated Antibody Cocktail & Wash

5. Reconstitute the detection antibody by adding 1.4 ml of Sample Diluent to the tube. Spin briefly.

6. Add 80 µl of the detection antibody cocktail to each well. Incubate at room temperature for 1-2 hour. Longer incubation time is preferable for higher signals

7. Decant the samples from each well, and wash 5 times (5 mins each) with 150 µl of 1X Wash Buffer I and then 2 times with 150 µl of 1x Wash Buffer II at room temperature with gentle shaking. Completely remove wash buffer in each wash step.

D. Incubation with Cy3 Equivalent Dye-Streptavidin & Wash

8. After briefly spinning down, add 1.4 ml of Sample Diluent to Cy3 equivalent dye-conjugated streptavidin tube. Mix gently.

9. Add 80 µl of Cy3 equivalent dye-conjugated streptavidin to each well. Cover the device with aluminum foil to avoid exposure to light or incubate in dark room. Incubate at room temperature for 1 hour.

10. Decant the samples from each well, and wash 5 times (5 mins each) with 150 µl of 1X Wash Buffer I at room temperature with gentle shaking. Completely remove wash buffer in each wash step.

E. Fluorescence Detection

11. Disassemble the device by pushing clips outward from the slide side. Carefully remove the slide from the gasket.

12. Place the slide in the Slide Washer/Dryer (a 4-slide holder/centrifuge tube), add enough 1x Wash Buffer I (about 30 ml) to cover the whole slide, and then gently shake at room temperature for 15 minutes. Decant Wash Buffer I. Wash with 1x Wash Buffer II (about 30 ml) and gently shake at room temperature for 5 minutes.

13. Remove water droplets completely by gently applying suction with a pipette to remove water droplets. Do not touch the array, only the sides.

14. Imaging: The signals can be visualized through use of a laser scanner equipped with a Cy3 wavelength (green channel) such as Axon GenePix or Innopsys Innoscan.

F. Data Analysis

15. Data extraction can be done using the GAL file that is specific for this array along with the microarray analysis software (GenePix, ScanArray Express, ArrayVision, MicroVigene, etc.).

Text S3 STROBE-MR: Guidelines for strengthening the reporting of Mendelian randomization studies[12]

1. TITLE and ABSTRACT

Indicate Mendelian randomization as the study's design in the title and/or the abstract.

Title; Abstract – Methods

INTRODUCTION

2. Background

Explain the scientific background and rationale for the reported study. Is causality between exposure and outcome plausible? Justify why MR is a helpful method to address the study question.

Introduction – Paragraphs 1-2.

3. Objectives

State specific objectives clearly, including pre-specified causal hypotheses (if any).

Introduction – Paragraph 3.

METHODS

4. Study design and data sources

Present key elements of study design early in the paper. Consider including a table listing sources of data for all phases of the study.

Methods – Publicly available GWAS summary data for 2-sample MR analyses - Paragraphs 1; Table S1.

5. Assumptions

Explicitly state assumptions for the main analysis (e.g. relevance, exclusion, independence, homogeneity) as well assumptions for any additional or sensitivity analysis.

Methods –Two-sample Mendelian Randomization – Paragraph 1.

6. Statistical methods: main analysis

Describe statistical methods and statistics used.

a) Describe how quantitative variables were handled in the analyses (i.e., scale, units, model).

N/A. (Two-sample design)

b) Describe the process for identifying genetic variants and weights to be included in the analyses (i.e, independence and model)

Methods – Selection of genetic instruments – Paragraph 1.

c) Describe the MR estimator, e.g. two-stage least squares, Wald ratio, and related statistics. Detail the included covariates and, in case of two-sample MR, whether the same covariate set was used for adjustment in the two samples.

Methods –Two-sample Mendelian Randomization – Paragraph 1-2; Table S1.

d) If applicable, say how multiple testing was dealt with.

Methods –Two-sample Mendelian Randomization – Paragraph 1.

7. Assessment of assumptions

Describe any methods used to assess the assumptions or justify their validity.

Methods –Two-sample Mendelian Randomization – Paragraph 2-3.

8. Sensitivity analyses

Describe any sensitivity analyses or additional analyses performed.

Methods –Two-sample Mendelian Randomization – Paragraph 2-3.

9. Software and pre-registration

a) Name statistical software and package(s), including version and settings used.

Methods – Statistical Analysis – Paragraph 1.

b) State whether the study protocol and details were pre-registered (as well as when and where).

N/A.

RESULTS

10. Descriptive data

For two-sample Mendelian randomization:

Provide information on extent of sample overlap between the exposure and outcome data sources.

Methods – Publicly available GWAS summary data for 2-sample MR analyses – Paragraph 1.

11. Main results

a) Report the associations between genetic variant and exposure, and between genetic variant and outcome, preferably on an interpretable scale (e.g. comparing 25th and 75th percentile of allele count or genetic risk score, if individual-level data available).

   Table S3.

b) Report causal effect estimate between exposure and outcome, and the measures of uncertainty from the MR analysis. Use an intuitive scale, such as odds ratio, or relative risk, per standard deviation difference.

   Results Table 2-3; Table S7-9.

c) If relevant, consider translating estimates of relative risk into absolute risk for a meaningful time-period.

   N/A.

d) Consider any plots to visualize results (e.g. forest plot, scatterplot of associations between genetic variants and outcome versus between genetic variants and exposure).

   Result Figure 2; Figure S3.

12. Assessment of assumptions

a) Assess the validity of the assumptions.

Results Table 2; Figure S3.

b) Report any additional statistics (e.g., assessments of heterogeneity, such as I^2, Q statistic).

Results – paragraph 3.

13. Sensitivity and additional analyses

a) Use sensitivity analyses to assess the robustness of the main results to violations of the assumptions.

Results – paragraph 3.

b) Report results from other sensitivity analyses (e.g., replication study with different dataset, analyses of subgroups, validation of instrument(s), simulations, etc.)

Results – Paragraph 4-5 (External replication, secondary outcome with similar etiology).

c) Report any assessment of direction of causality (e.g., bidirectional MR).

N/A.

DISCUSSION

14. Key results

Summarize key results with reference to study objectives.

Discussion – Paragraph 1.

15. Limitations

Discuss limitations of the study, taking into account the validity of the MR assumptions, other sources of potential bias, and imprecision. Discuss both direction and magnitude of any potential bias, and any efforts to address them.

Discussion – Strengths and Limitations – Paragraph 2.

16. Interpretation

a) Give a cautious overall interpretation of results considering objectives and limitations. Compare with results from other relevant studies.

Discussion – Paragraph 1-2.

b) Discuss underlying biological mechanisms that could be modelled by using the genetic variants to assess the relationship between the exposure and the outcome.

NA.

c) Discuss whether the results have clinical or policy relevance, and whether interventions could have the same size effect.

Discussion – Paragraph 2-4.

17. Generalizability

Discuss the generalizability of the study results (a) to other populations (i.e. external validity), (b) across other exposure periods/timings, and (c) across other levels of exposure.

Discussion – Strengths and limitations.

OTHER INFORMATION

18. Funding

Give the source of funding and the role of the funders for the present study and, if applicable, for the original study or studies on which the present article is based.

Funding

19. Data and data sharing

Present data used to perform all analyses or report where and how the data can be accessed. State whether statistical code is publicly accessible and if so, where.

Acknowledge; Table S1.

20. Conflicts of Interest

All authors should declare all potential conflicts of interest.

Conflicts of Interest

Text S4 Concept of mediation analysis

Mediation analysis determines **if potential mediators mediate the association** between independent variable with dependent variable and **quantifies their contribution**[113]. Several criteria should be satisfied in mediation analysis: 1) Independent variable (X) must be significantly associated with dependent variable (Y) (The effect from X to Y is *c*); 2) X must be significantly associated with mediator (M) (The effect from X to M is *a*); 3) M must be significantly associated with Y (The effect from M to Y is *b*). *c*' is the effect of from X to Y bypassing the investigated mediator, calculated through adjustment for the investigated mediator in model. Based on the design of mediation analysis (as shown in the following Figure), the total effect of X on Y (*c*) was divided into direct effect (*c'*, the effect that was not transmitted by selected mediators) and indirect effect (*a*b*, the effect that was mediated by the investigated mediator) ($c=a*b+c'$). Proportion mediated (PM, %) was defined as indirect effect/total effect (PM= $a*b/c$).

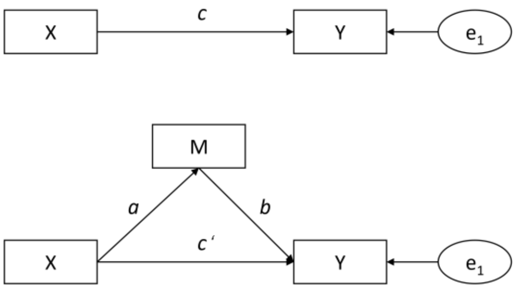

Figure Frame of mediation analysis

In the present study, we used mediation analysis to assess the extent to which the association of GP73 with CAD was mediated by two mediators (LDL-c and HbA1c). The total effect [log(odds ratio, OR)] of GP73 on CAD was 0.107 [log(1.11)]. The effect of GP73 on LDL-c was 0.099, and LDL-c was associated with CAD [log(OR)=log(1.58)=0.456]. Hence, the mediated effect of LDL-c was 0.099×0.456=0.0451. The mediated proportion was 0.0451/0.107 = 42.1%.

Text S5 Information on experimental procedures

All experimental procedures involving animals were performed according to the Guide for the Care and Use of Laboratory Animals (NIH Publication, 8th edition, 2011) and were approved by the ethic committees of Sun Yat-sen University.

ApoE−/− mice (8weeks; male; Vital River, Beijing, China) were housed in specific pathogen-free conditions and randomly allocated into either high-fat diet (15% lard, 20% sugar, and 1.2% Cholesterol; n = 5) or normal diet group (n = 5) for 12 weeks. High-fat diet was used to induce atherosclerosis and normal diet group served as the control group. After the modeling is completed, mice were weighted then anesthetized by intraperitoneal injection of 1% pentobarbital sodium. Regular vital signs (respiratory rate and pulse) but absence of toe-pinch reflex indicated the adequacy of anesthesia. Blood samples was collected for extracting Serum specimens were extracted from whole blood samples and stored at -80℃ until measurement. Aortic tissues were isolated for Western blotting and immunofluorescence staining. The aortic roots were processed into optimum cutting temperature compound (OCT) and sequentially sectioned into 8 μm-thick with a cryostat. Enzymatic kits (Jiancheng Biotechnology, Nanjing, China) were implemented for measurements of plasma total cholesterol, low-density lipoprotein cholesterol and high-density lipoprotein cholesterol.

Text S6 MEGASTROKE CONSORTIUM

Rainer Malik 1, Ganesh Chauhan 2, Matthew Traylor 3, Muralidharan Sargurupremraj 4,5, Yukinori Okada 6,7,8, Aniket Mishra 4,5, Loes Rutten-Jacobs 3, Anne-Katrin Giese 9, Sander W van der Laan 10, Solveig Gretarsdottir 11, Christopher D Anderson 12,13,14,14, Michael Chong 15, Hieab HH Adams 16,17, Tetsuro Ago 18, Peter Almgren 19, Philippe Amouyel 20,21, Hakan Ay 22,13, Traci M Bartz 23, Oscar R Benavente 24, Steve Bevan 25, Giorgio B Boncoraglio 26, Robert D Brown, Jr. 27, Adam S Butterworth 28,29, Caty Carrera 30,31, Cara L Carty 32,33, Daniel I Chasman 34,35, Wei-Min Chen 36, John W Cole 37, Adolfo Correa 38, Ioana Cotlarciuc 39, Carlos Cruchaga 40,41, John Danesh 28,42,43,44, Paul IW de Bakker 45,46, Anita L DeStefano 47,48, Marcel den Hoed 49, Qing Duan 50, Stefan T Engelter 51,52, Guido J Falcone 53,54, Rebecca F Gottesman 55, Raji P Grewal 56, Vilmundur Gudnason 57,58, Stefan Gustafsson 59, Jeffrey Haessler 60, Tamara B Harris 61, Ahamad Hassan 62, Aki S Havulinna 63,64, Susan R Heckbert 65, Elizabeth G Holliday 66,67, George Howard 68, Fang-Chi Hsu 69, Hyacinth I Hyacinth 70, M Arfan Ikram 16, Erik Ingelsson 71,72, Marguerite R Irvin 73, Xueqiu Jian 74, Jordi Jiménez-Conde 75, Julie A Johnson 76,77, J Wouter Jukema 78, Masahiro Kanai 6,7,79, Keith L Keene 80,81, Brett M Kissela 82, Dawn O Kleindorfer 82, Charles Kooperberg 60, Michiaki Kubo 83, Leslie A Lange 84, Carl D Langefeld 85, Claudia Langenberg 86, Lenore J Launer 87, Jin-Moo Lee 88, Robin Lemmens 89,90, Didier Leys 91, Cathryn M Lewis 92,93, Wei-Yu Lin 28,94, Arne G Lindgren 95,96, Erik Lorentzen 97, Patrik K Magnusson 98, Jane Maguire 99, Ani Manichaikul 36, Patrick F McArdle 100, James F Meschia 101, Braxton D Mitchell 100,102, Thomas H Mosley 103,104, Michael A Nalls 105,106, Toshiharu Ninomiya 107, Martin J O'Donnell 15,108, Bruce M Psaty 109,110,111,112, Sara L Pulit 113,45, Kristiina Rannikmäe 114,115, Alexander P Reiner 65,116, Kathryn M Rexrode 117, Kenneth Rice 118, Stephen S Rich 36, Paul M Ridker 34,35, Natalia S Rost 9,13, Peter M Rothwell 119, Jerome I Rotter 120,121, Tatjana Rundek 122, Ralph L Sacco 122, Saori Sakaue 7,123, Michele M Sale 124, Veikko Salomaa 63, Bishwa R Sapkota 125, Reinhold Schmidt 126, Carsten O Schmidt 127, Ulf Schminke 128, Pankaj Sharma 39, Agnieszka Slowik 129, Cathie LM Sudlow 114,115, Christian Tanislav 130, Turgut Tatlisumak 131,132, Kent D Taylor 120,121, Vincent NS Thijs 133,134, Gudmar Thorleifsson 11, Unnur Thorsteinsdottir 11, Steffen Tiedt 1, Stella Trompet 135, Christophe Tzourio 5,136,137, Cornelia M van Duijn 138,139,

Matthew Walters 140, Nicholas J Wareham 86, Sylvia Wassertheil-Smoller 141, James G Wilson 142, Kerri L Wiggins 109, Qiong Yang 47, Salim Yusuf 15, Najaf Amin 16, Hugo S Aparicio 185,48, Donna K Arnett 186, John Attia 187, Alexa S Beiser 47,48, Claudine Berr 188, Julie E Buring 34,35, Mariana Bustamante 189, Valeria Caso 190, Yu-Ching Cheng 191, Seung Hoan Choi 192,48, Ayesha Chowhan 185,48, Natalia Cullell 31, Jean-François Dartigues 193,194, Hossein Delavaran 95,96, Pilar Delgado 195, Marcus Dörr 196,197, Gunnar Engström 19, Ian Ford 198, Wander S Gurpreet 199, Anders Hamsten 200,201, Laura Heitsch 202, Atsushi Hozawa 203, Laura Ibanez 204, Andreea Ilinca 95,96, Martin Ingelsson 205, Motoki Iwasaki 206, Rebecca D Jackson 207, Katarina Jood 208, Pekka Jousilahti 63, Sara Kaffashian 4,5, Lalit Kalra 209, Masahiro Kamouchi 210, Takanari Kitazono 211, Olafur Kjartansson 212, Manja Kloss 213, Peter J Koudstaal 214, Jerzy Krupinski 215, Daniel L Labovitz 216, Cathy C Laurie 118, Christopher R Levi 217, Linxin Li 218, Lars Lind 219, Cecilia M Lindgren 220,221, Vasileios Lioutas 222,48, Yong Mei Liu 223, Oscar L Lopez 224, Hirata Makoto 225, Nicolas Martinez-Majander 172, Koichi Matsuda 225, Naoko Minegishi 203, Joan Montaner 226, Andrew P Morris 227,228, Elena Muiño 31, Martina Müller-Nurasyid 229,230,231, Bo Norrving 95,96, Soichi Ogishima 203, Eugenio A Parati 232, Leema Reddy Peddareddygari 56, Nancy L Pedersen 98,233, Joanna Pera 129, Markus Perola 63,234, Alessandro Pezzini 235, Silvana Pileggi 236, Raquel Rabionet 237, Iolanda Riba-Llena 30, Marta Ribasés 238, Jose R Romero 185,48, Jaume Roquer 239,240, Anthony G Rudd 241,242, Antti-Pekka Sarin 243,244, Ralhan Sarju 199, Chloe Sarnowski 47,48, Makoto Sasaki 245, Claudia L Satizabal 185,48, Mamoru Satoh 245, Naveed Sattar 246, Norie Sawada 206, Gerli Sibolt 172, Ásgeir Sigurdsson 247, Albert Smith 248, Kenji Sobue 245, Carolina Soriano-Tárraga 240, Tara Stanne 249, O Colin Stine 250, David J Stott 251, Konstantin Strauch 229,252, Takako Takai 203, Hideo Tanaka 253,254, Kozo Tanno 245, Alexander Teumer 255, Liisa Tomppo 172, Nuria P Torres-Aguila 31, Emmanuel Touze 256,257, Shoichiro Tsugane 206, Andre G Uitterlinden 258, Einar M Valdimarsson 259, Sven J van der Lee 16, Henry Völzke 255, Kenji Wakai 253, David Weir 260, Stephen R Williams 261, Charles DA Wolfe 241,242, Quenna Wong 118, Huichun Xu 191, Taiki Yamaji 206, Dharambir K Sanghera 125,169,170, Olle Melander 19, Christina Jern 171, Daniel Strbian 172,173, Israel Fernandez-Cadenas 31,30, W T Longstreth, Jr 174,65, Arndt Rolfs 175, Jun Hata 107, Daniel Woo 82, Jonathan Rosand 12,13,14, Guillaume Pare 15, Jemma C

Hopewell 176, Danish Saleheen 177, Kari Stefansson 11,178, Bradford B Worrall 179, Steven J Kittner 37, Sudha Seshadri 180,48, Myriam Fornage 74,181, Hugh S Markus 3, Joanna MM Howson 28, Yoichiro Kamatani 6,182, Stephanie Debette 4,5, Martin Dichgans 1,183,184

1 Institute for Stroke and Dementia Research (ISD), University Hospital, LMU Munich, Munich, Germany

2 Centre for Brain Research, Indian Institute of Science, Bangalore, India

3 Stroke Research Group, Division of Clinical Neurosciences, University of Cambridge, UK

4 INSERM U1219 Bordeaux Population Health Research Center, Bordeaux, France

5 University of Bordeaux, Bordeaux, France

6 Laboratory for Statistical Analysis, RIKEN Center for Integrative Medical Sciences, Yokohama, Japan

7 Department of Statistical Genetics, Osaka University Graduate School of Medicine, Osaka, Japan

8 Laboratory of Statistical Immunology, Immunology Frontier Research Center (WPI-IFReC), Osaka University, Suita, Japan.

9 Department of Neurology, Massachusetts General Hospital, Harvard Medical School, Boston, MA, USA

10 Laboratory of Experimental Cardiology, Division of Heart and Lungs, University Medical Center Utrecht, University of Utrecht, Utrecht,Netherlands

11 deCODE genetics/AMGEN inc, Reykjavik, Iceland

12 Center for Genomic Medicine, Massachusetts General Hospital (MGH), Boston, MA, USA

13 J. Philip Kistler Stroke Research Center, Department of Neurology, MGH, Boston, MA, USA

14 Program in Medical and Population Genetics, Broad Institute, Cambridge, MA, USA

15 Population Health Research Institute, McMaster University, Hamilton, Canada

16 Department of Epidemiology, Erasmus University Medical Center, Rotterdam, Netherlands

17 Department of Radiology and Nuclear Medicine, Erasmus University Medical Center, Rotterdam, Netherlands

18 Department of Medicine and Clinical Science, Graduate School of Medical Sciences, Kyushu

University, Fukuoka, Japan

19 Department of Clinical Sciences, Lund University, Malmö, Sweden

20 Univ. Lille, Inserm, Institut Pasteur de Lille, LabEx DISTALZ-UMR1167, Risk factors and molecular determinants of aging-related diseases, F-59000 Lille, France

21 Centre Hosp. Univ Lille, Epidemiology and Public Health Department, F-59000 Lille, France

22 AA Martinos Center for Biomedical Imaging, Department of Radiology, Massachusetts General Hospital, Harvard Medical School, Boston, MA, USA

23 Cardiovascular Health Research Unit, Departments of Biostatistics and Medicine, University of Washington, Seattle, WA, USA

24 Division of Neurology, Faculty of Medicine, Brain Research Center, University of British Columbia, Vancouver, Canada

25 School of Life Science, University of Lincoln, Lincoln, UK

26 Department of Cerebrovascular Diseases, Fondazione IRCCS Istituto Neurologico "Carlo Besta", Milano, Italy

27 Department of Neurology, Mayo Clinic Rochester, Rochester, MN, USA

28 MRC/BHF Cardiovascular Epidemiology Unit, Department of Public Health and Primary Care, University of Cambridge, Cambridge, UK

29 The National Institute for Health Research Blood and Transplant Research Unit in Donor Health and Genomics, University of Cambridge, UK

30 Neurovascular Research Laboratory, Vall d'Hebron Institut of Research, Neurology and Medicine Departments-Universitat Autònoma de Barcelona, Vall d’Hebrón Hospital, Barcelona, Spain

31 Stroke Pharmacogenomics and Genetics, Fundacio Docència i Recerca MutuaTerrassa, Terrassa, Spain

32 Children's Research Institute, Children's National Medical Center, Washington, DC, USA

33 Center for Translational Science, George Washington University, Washington, DC, USA

34 Division of Preventive Medicine, Brigham and Women's Hospital, Boston, MA, USA

35 Harvard Medical School, Boston, MA, USA

36 Center for Public Health Genomics, Department of Public Health Sciences, University of Virginia,

Charlottesville, VA, USA

37 Department of Neurology, University of Maryland School of Medicine and Baltimore VAMC, Baltimore, MD, USA

38 Departments of Medicine, Pediatrics and Population Health Science, University of Mississippi Medical Center, Jackson, MS, USA

39 Institute of Cardiovascular Research, Royal Holloway University of London, UK & Ashford and St Peters Hospital, Surrey UK

40 Department of Psychiatry,The Hope Center Program on Protein Aggregation and Neurodegeneration (HPAN),Washington University, School of Medicine, St. Louis, MO, USA

41 Department of Developmental Biology, Washington University School of Medicine, St. Louis, MO, USA

42 NIHR Blood and Transplant Research Unit in Donor Health and Genomics, Department of Public Health and Primary Care, University of Cambridge, Cambridge, UK

43 Wellcome Trust Sanger Institute, Wellcome Trust Genome Campus, Hinxton, Cambridge, UK

44 British Heart Foundation, Cambridge Centre of Excellence, Department of Medicine, University of Cambridge, Cambridge, UK

45 Department of Medical Genetics, University Medical Center Utrecht, Utrecht, Netherlands

46 Department of Epidemiology, Julius Center for Health Sciences and Primary Care, University Medical Center Utrecht, Utrecht, Netherlands

47 Boston University School of Public Health, Boston, MA, USA

48 Framingham Heart Study, Framingham, MA, USA

49 Department of Immunology, Genetics and Pathology and Science for Life Laboratory, Uppsala University, Uppsala, Sweden

50 Department of Genetics, University of North Carolina, Chapel Hill, NC, USA

51 Department of Neurology and Stroke Center, Basel University Hospital, Switzerland

52 Neurorehabilitation Unit, University and University Center for Medicine of Aging and Rehabilitation Basel, Felix Platter Hospital, Basel, Switzerland

53 Department of Neurology, Yale University School of Medicine, New Haven, CT, USA

54 Program in Medical and Population Genetics, The Broad Institute of Harvard and MIT, Cambridge, MA, USA

55 Department of Neurology, Johns Hopkins University School of Medicine, Baltimore, MD, USA

56 Neuroscience Institute, SF Medical Center, Trenton, NJ, USA

57 Icelandic Heart Association Research Institute, Kopavogur, Iceland

58 University of Iceland, Faculty of Medicine, Reykjavik, Iceland

59 Department of Medical Sciences, Molecular Epidemiology and Science for Life Laboratory, Uppsala University, Uppsala, Sweden

60 Division of Public Health Sciences, Fred Hutchinson Cancer Research Center, Seattle, WA, USA

61 Laboratory of Epidemiology and Population Science, National Institute on Aging, National Institutes of Health, Bethesda, MD, USA

62 Department of Neurology, Leeds General Infirmary, Leeds Teaching Hospitals NHS Trust, Leeds, UK

63 National Institute for Health and Welfare, Helsinki, Finland

64 FIMM - Institute for Molecular Medicine Finland, Helsinki, Finland

65 Department of Epidemiology, University of Washington, Seattle, WA, USA

66 Public Health Stream, Hunter Medical Research Institute, New Lambton, Australia

67 Faculty of Health and Medicine, University of Newcastle, Newcastle, Australia

68 School of Public Health, University of Alabama at Birmingham, Birmingham, AL, USA

69 Department of Biostatistical Sciences, Wake Forest School of Medicine, Winston-Salem, NC, USA

70 Aflac Cancer and Blood Disorder Center, Department of Pediatrics, Emory University School of Medicine, Atlanta, GA, USA

71 Department of Medicine, Division of Cardiovascular Medicine, Stanford University School of Medicine, CA, USA

72 Department of Medical Sciences, Molecular Epidemiology and Science for Life Laboratory, Uppsala University, Uppsala, Sweden

73 Epidemiology, School of Public Health, University of Alabama at Birmingham, USA

74 Brown Foundation Institute of Molecular Medicine, University of Texas Health Science Center at

Houston, Houston, TX, USA

75 Neurovascular Research Group (NEUVAS), Neurology Department, Institut Hospital del Mar d'Investigació Mèdica, Universitat Autònoma de Barcelona, Barcelona, Spain

76 Department of Pharmacotherapy and Translational Research and Center for Pharmacogenomics, University of Florida, College of Pharmacy, Gainesville, FL, USA

77 Division of Cardiovascular Medicine, College of Medicine, University of Florida, Gainesville, FL, USA

78 Department of Cardiology, Leiden University Medical Center, Leiden, the Netherlands

79 Program in Bioinformatics and Integrative Genomics, Harvard Medical School, Boston, MA, USA

80 Department of Biology, East Carolina University, Greenville, NC, USA

81 Center for Health Disparities, East Carolina University, Greenville, NC, USA

82 University of Cincinnati College of Medicine, Cincinnati, OH, USA

83 RIKEN Center for Integrative Medical Sciences, Yokohama, Japan

84 Department of Medicine, University of Colorado Denver, Anschutz Medical Campus, Aurora, CO, USA

85 Center for Public Health Genomics and Department of Biostatistical Sciences, Wake Forest School of Medicine, Winston-Salem, NC, USA

86 MRC Epidemiology Unit, University of Cambridge School of Clinical Medicine, Institute of Metabolic Science, Cambridge Biomedical Campus, Cambridge, UK

87 Intramural Research Program, National Institute on Aging, National Institutes of Health, Bethesda, MD, USA

88 Department of Neurology, Radiology, and Biomedical Engineering, Washington University School of Medicine, St. Louis, MO, USA

89 KU Leuven – University of Leuven, Department of Neurosciences, Experimental Neurology, Leuven, Belgium

90 VIB Center for Brain & Disease Research, University Hospitals Leuven, Department of Neurology, Leuven, Belgium

91 Univ.-Lille, INSERM U 1171. CHU Lille. Lille, France

92 Department of Medical and Molecular Genetics, King's College London, London, UK

93 SGDP Centre, Institute of Psychiatry, Psychology & Neuroscience, King's College London, London, UK

94 Northern Institute for Cancer Research, Paul O'Gorman Building, Newcastle University, Newcastle, UK

95 Department of Clinical Sciences Lund, Neurology, Lund University, Lund, Sweden

96 Department of Neurology and Rehabilitation Medicine, Skåne University Hospital, Lund, Sweden

97 Bioinformatics Core Facility, University of Gothenburg, Gothenburg, Sweden

98 Department of Medical Epidemiology and Biostatistics, Karolinska Institutet, Stockholm, Sweden

99 University of Technology Sydney, Faculty of Health, Ultimo, Australia

100 Department of Medicine, University of Maryland School of Medicine, MD, USA

101 Department of Neurology, Mayo Clinic, Jacksonville, FL, USA

102 Geriatrics Research and Education Clinical Center, Baltimore Veterans Administration Medical Center, Baltimore, MD, USA

103 Division of Geriatrics, School of Medicine, University of Mississippi Medical Center, Jackson, MS, USA

104 Memory Impairment and Neurodegenerative Dementia Center, University of Mississippi Medical Center, Jackson, MS, USA

105 Laboratory of Neurogenetics, National Institute on Aging, National institutes of Health, Bethesda, MD, USA

106 Data Tecnica International, Glen Echo MD, USA

107 Department of Epidemiology and Public Health, Graduate School of Medical Sciences, Kyushu University, Fukuoka, Japan

108 Clinical Research Facility, Department of Medicine, NUI Galway, Galway, Ireland

109 Cardiovascular Health Research Unit, Department of Medicine, University of Washington, Seattle, WA, USA

110 Department of Epidemiology, University of Washington, Seattle, WA

111 Department of Health Services, University of Washington, Seattle, WA, USA

112 Kaiser Permanente Washington Health Research Institute, Seattle, WA, USA

113 Brain Center Rudolf Magnus, Department of Neurology, University Medical Center Utrecht, Utrecht, The Netherlands

114 Usher Institute of Population Health Sciences and Informatics, University of Edinburgh, Edinburgh, UK

115 Centre for Clinical Brain Sciences, University of Edinburgh, Edinburgh, UK

116 Fred Hutchinson Cancer Research Center, University of Washington, Seattle, WA, USA

117 Department of Medicine, Brigham and Women's Hospital, Boston, MA, USA

118 Department of Biostatistics, University of Washington, Seattle, WA, USA

119 Nuffield Department of Clinical Neurosciences, University of Oxford, UK

120 Institute for Translational Genomics and Population Sciences, Los Angeles Biomedical Research Institute at Harbor-UCLA Medical Center, Torrance, CA, USA

121 Division of Genomic Outcomes, Department of Pediatrics, Harbor-UCLA Medical Center, Torrance, CA, USA

122 Department of Neurology, Miller School of Medicine, University of Miami, Miami, FL, USA

123 Department of Allergy and Rheumatology, Graduate School of Medicine, the University of Tokyo, Tokyo, Japan

124 Center for Public Health Genomics, University of Virginia, Charlottesville, VA, USA

125 Department of Pediatrics, College of Medicine, University of Oklahoma Health Sciences Center, Oklahoma City, OK, USA

126 Department of Neurology, Medical University of Graz, Graz, Austria

127 University Medicine Greifswald, Institute for Community Medicine, SHIP-KEF, Greifswald, Germany

128 University Medicine Greifswald, Department of Neurology, Greifswald, Germany

129 Department of Neurology, Jagiellonian University, Krakow, Poland

130 Department of Neurology, Justus Liebig University, Giessen, Germany

131 Department of Clinical Neurosciences/Neurology, Institute of Neuroscience and Physiology, Sahlgrenska Academy at University of Gothenburg, Gothenburg, Sweden

132 Sahlgrenska University Hospital, Gothenburg, Sweden

133 Stroke Division, Florey Institute of Neuroscience and Mental Health, University of Melbourne, Heidelberg, Australia

134 Austin Health, Department of Neurology, Heidelberg, Australia

135 Department of Internal Medicine, Section Gerontology and Geriatrics, Leiden University Medical Center, Leiden, the Netherlands

136 INSERM U1219, Bordeaux, France

137 Department of Public Health, Bordeaux University Hospital, Bordeaux, France

138 Genetic Epidemiology Unit, Department of Epidemiology, Erasmus University Medical Center Rotterdam, Netherlands

139 Center for Medical Systems Biology, Leiden, Netherlands

140 School of Medicine, Dentistry and Nursing at the University of Glasgow, Glasgow, UK

141 Department of Epidemiology and Population Health, Albert Einstein College of Medicine, NY, USA

142 Department of Physiology and Biophysics, University of Mississippi Medical Center, Jackson, MS, USA

143 A full list of members and affiliations appears in the Supplementary Note

144 Department of Human Genetics, McGill University, Montreal, Canada

145 Department of Pathophysiology, Institute of Biomedicine and Translation Medicine, University of Tartu, Tartu, Estonia

146 Department of Cardiac Surgery, Tartu University Hospital, Tartu, Estonia

147 Clinical Gene Networks AB,Stockholm, Sweden

148 Department of Genetics and Genomic Sciences, The Icahn Institute for Genomics and Multiscale iology Icahn School of Medicine at Mount Sinai, New York, NY , USA

149 Department of Pathophysiology, Institute of Biomedicine and Translation Medicine, University of Tartu, Biomeedikum, Tartu, Estonia

150 Integrated Cardio Metabolic Centre, Department of Medicine, Karolinska Institutet, Karolinska Universitetssjukhuset, Huddinge, Sweden.

151 Clinical Gene Networks AB, Stockholm, Sweden

152 Sorbonne Universités, UPMC Univ. Paris 06, INSERM, UMR_S 1166, Team Genomics & Pathophysiology of Cardiovascular Diseases, Paris, France

153 ICAN Institute for Cardiometabolism and Nutrition, Paris, France

154 Department of Biomedical Engineering, University of Virginia, Charlottesville, VA, USA

155 Group Health Research Institute, Group Health Cooperative, Seattle, WA, USA

156 Seattle Epidemiologic Research and Information Center, VA Office of Research and Development, Seattle, WA, USA

157 Cardiovascular Research Center, Massachusetts General Hospital, Boston, MA, USA

158 Department of Medical Research, Bærum Hospital, Vestre Viken Hospital Trust, Gjettum, Norway

159 Saw Swee Hock School of Public Health, National University of Singapore and National University Health System, Singapore

160 National Heart and Lung Institute, Imperial College London, London, UK

161 Department of Gene Diagnostics and Therapeutics, Research Institute, National Center for Global Health and Medicine, Tokyo, Japan

162 Department of Epidemiology, Tulane University School of Public Health and Tropical Medicine, New Orleans, LA, USA

163 Department of Cardiology,University Medical Center Groningen, University of Groningen, Netherlands

164 MRC-PHE Centre for Environment and Health, School of Public Health, Department of Epidemiology and Biostatistics, Imperial College London, London, UK

165 Department of Epidemiology and Biostatistics, Imperial College London, London, UK

166 Department of Cardiology, Ealing Hospital NHS Trust, Southall, UK

167 National Heart, Lung and Blood Research Institute, Division of Intramural Research, Population Sciences Branch, Framingham, MA, USA

168 A full list of members and affiliations appears at the end of the manuscript

169 Department of Phamaceutical Sciences, Collge of Pharmacy, University of Oklahoma Health Sciences Center, Oklahoma City, OK, USA

170 Oklahoma Center for Neuroscience, Oklahoma City, OK, USA

171 Department of Pathology and Genetics, Institute of Biomedicine, The Sahlgrenska Academy at University of Gothenburg, Gothenburg, Sweden

172 Department of Neurology, Helsinki University Hospital, Helsinki, Finland

173 Clinical Neurosciences, Neurology, University of Helsinki, Helsinki, Finland

174 Department of Neurology, University of Washington, Seattle, WA, USA

175 Albrecht Kossel Institute, University Clinic of Rostock, Rostock, Germany

176 Clinical Trial Service Unit and Epidemiological Studies Unit, Nuffield Department of Population Health, University of Oxford, Oxford, UK

177 Department of Genetics, Perelman School of Medicine, University of Pennsylvania, PA, USA

178 Faculty of Medicine, University of Iceland, Reykjavik, Iceland

179 Departments of Neurology and Public Health Sciences, University of Virginia School of Medicine, Charlottesville, VA, USA

180 Department of Neurology, Boston University School of Medicine, Boston, MA, USA

181 Human Genetics Center, University of Texas Health Science Center at Houston, Houston, TX, USA

182 Center for Genomic Medicine, Kyoto University Graduate School of Medicine, Kyoto, Japan

183 Munich Cluster for Systems Neurology (SyNergy), Munich, Germany

184 German Center for Neurodegenerative Diseases (DZNE), Munich, Germany

185 Boston University School of Medicine, Boston, MA, USA

186 University of Kentucky College of Public Health, Lexington, KY, USA

187 University of Newcastle and Hunter Medical Research Institute, New Lambton, Australia

188 Univ. Montpellier, Inserm, U1061, Montpellier, France

189 Centre for Research in Environmental Epidemiology, Barcelona, Spain

190 Department of Neurology, Università degli Studi di Perugia, Umbria, Italy

191 Department of Medicine, University of Maryland School of Medicine, Baltimore, MD, USA

192 Broad Institute, Cambridge, MA, USA

193 Univ. Bordeaux, Inserm, Bordeaux Population Health Research Center, UMR 1219, Bordeaux,

France

194 Bordeaux University Hospital, Department of Neurology, Memory Clinic, Bordeaux, France

195 Neurovascular Research Laboratory. Vall d'Hebron Institut of Research, Neurology and Medicine Departments-Universitat Autònoma de Barcelona. Vall d'Hebrón Hospital, Barcelona, Spain

196 University Medicine Greifswald, Department of Internal Medicine B, Greifswald, Germany

197 DZHK, Greifswald, Germany

198 Robertson Center for Biostatistics, University of Glasgow, Glasgow, UK

199 Hero DMC Heart Institute, Dayanand Medical College & Hospital, Ludhiana, India

200 Atherosclerosis Research Unit, Department of Medicine Solna, Karolinska Institutet, Stockholm, Sweden

201 Karolinska Institutet, Stockholm, Sweden

202 Division of Emergency Medicine, and Department of Neurology, Washington University School of Medicine, St. Louis, MO, USA

203 Tohoku Medical Megabank Organization, Sendai, Japan

204 Department of Psychiatry, Washington University School of Medicine, St. Louis, MO, USA

205 Department of Public Health and Caring Sciences / Geriatrics, Uppsala University, Uppsala, Sweden

206 Epidemiology and Prevention Group, Center for Public Health Sciences, National Cancer Center, Tokyo, Japan

207 Department of Internal Medicine and the Center for Clinical and Translational Science, The Ohio State University, Columbus, OH, USA

208 Institute of Neuroscience and Physiology, the Sahlgrenska Academy at University of Gothenburg, Goteborg, Sweden

209 Department of Basic and Clinical Neurosciences, King's College London, London, UK

210 Department of Health Care Administration and Management, Graduate School of Medical Sciences, Kyushu University, Japan

211 Department of Medicine and Clinical Science, Graduate School of Medical Sciences, Kyushu University, Japan

212 Landspitali National University Hospital, Departments of Neurology & Radiology, Reykjavik, Iceland

213 Department of Neurology, Heidelberg University Hospital, Germany

214 Department of Neurology, Erasmus University Medical Center

215 Hospital Universitari Mutua Terrassa, Terrassa (Barcelona), Spain

216 Albert Einstein College of Medicine, Montefiore Medical Center, New York, NY, USA

217 John Hunter Hospital, Hunter Medical Research Institute and University of Newcastle, Newcastle, NSW, Australia

218 Centre for Prevention of Stroke and Dementia, Nuffield Department of Clinical Neurosciences, University of Oxford, UK

219 Department of Medical Sciences, Uppsala University, Uppsala, Sweden

220 Genetic and Genomic Epidemiology Unit, Wellcome Trust Centre for Human Genetics, University of Oxford, Oxford, UK

221 The Wellcome Trust Centre for Human Genetics, Oxford, UK

222 Beth Israel Deaconess Medical Center, Boston, MA, USA

223 Wake Forest School of Medicine, Wake Forest, NC, USA

224 Department of Neurology, University of Pittsburgh, Pittsburgh, PA, USA

225 BioBank Japan, Laboratory of Clinical Sequencing, Department of Computational biology and medical Sciences, Graduate school of Frontier Sciences, The University of Tokyo, Tokyo, Japan

226 Neurovascular Research Laboratory, Vall d'Hebron Institut of Research, Neurology and Medicine Departments-Universitat Autònoma de Barcelona. Vall d’Hebrón Hospital, Barcelona, Spain

227 Department of Biostatistics, University of Liverpool, Liverpool, UK

228 Wellcome Trust Centre for Human Genetics, University of Oxford, Oxford, UK

229 Institute of Genetic Epidemiology, Helmholtz Zentrum München - German Research Center for Environmental Health, Neuherberg, Germany

230 Department of Medicine I, Ludwig-Maximilians-Universität, Munich, Germany

231 DZHK (German Centre for Cardiovascular Research), partner site Munich Heart Alliance, Munich, Germany

232 Department of Cerebrovascular Diseases, Fondazione IRCCS Istituto Neurologico “Carlo Besta”, Milano, Italy

233 Karolinska Institutet, MEB, Stockholm, Sweden

234 University of Tartu, Estonian Genome Center, Tartu, Estonia, Tartu, Estonia

235 Department of Clinical and Experimental Sciences, Neurology Clinic, University of Brescia, Italy

236 Translational Genomics Unit, Department of Oncology, IRCCS Istituto di Ricerche Farmacologiche Mario Negri, Milano, Italy

237 Department of Genetics, Microbiology and Statistics, University of Barcelona, Barcelona, Spain

238 Psychiatric Genetics Unit, Group of Psychiatry, Mental Health and Addictions, Vall d’Hebron Research Institute (VHIR), Universitat Autònoma de Barcelona, Biomedical Network Research Centre on Mental Health (CIBERSAM), Barcelona, Spain

239 Department of Neurology, IMIM-Hospital del Mar, and Universitat Autònoma de Barcelona, Spain

240 IMIM (Hospital del Mar Medical Research Institute), Barcelona, Spain

241 National Institute for Health Research Comprehensive Biomedical Research Centre, Guy's & St. Thomas' NHS Foundation Trust and King's College London, London, UK

242 Division of Health and Social Care Research, King's College London, London, UK

243 FIMM-Institute for Molecular Medicine Finland, Helsinki, Finland

244 THL-National Institute for Health and Welfare, Helsinki, Finland

245 Iwate Tohoku Medical Megabank Organization, Iwate Medical University, Iwate, Japan

246 BHF Glasgow Cardiovascular Research Centre, Faculty of Medicine, Glasgow, UK

247 deCODE Genetics/Amgen, Inc., Reykjavik, Iceland

248 Icelandic Heart Association, Reykjavik, Iceland

249 Institute of Biomedicine, the Sahlgrenska Academy at University of Gothenburg, Goteborg, Sweden

250 Department of Epidemiology, University of Maryland School of Medicine, Baltimore, MD, USA

251 Institute of Cardiovascular and Medical Sciences, Faculty of Medicine, University of Glasgow, Glasgow, UK

252 Chair of Genetic Epidemiology, IBE, Faculty of Medicine, LMU Munich, Germany

253 Division of Epidemiology and Prevention, Aichi Cancer Center Research Institute, Nagoya, Japan

254 Department of Epidemiology, Nagoya University Graduate School of Medicine, Nagoya, Japan

255 University Medicine Greifswald, Institute for Community Medicine, SHIP-KEF, Greifswald, Germany

256 Department of Neurology, Caen University Hospital, Caen, France

257 University of Caen Normandy, Caen, France

258 Department of Internal Medicine, Erasmus University Medical Center, Rotterdam, Netherlands

259 Landspitali University Hospital, Reykjavik, Iceland

260 Survey Research Center, University of Michigan, Ann Arbor, MI, USA

261 University of Virginia Department of Neurology, Charlottesville, VA, USA

References

1. Y. Zhan, I. K. Karlsson, R. Karlsson, et al. Exploring the Causal Pathway From Telomere Length to Coronary Heart Disease: A Network Mendelian Randomization Study. *CIRC RES* 2017;121(3):214-19. doi: 10.1161/CIRCRESAHA.116.310517 pmid:28515044.[(c) 2017 American Heart Association, Inc.:*2017-07-21].
2. M. Nikpay, A. Goel, H. H. Won, et al. A comprehensive 1,000 Genomes-based genome-wide association meta-analysis of coronary artery disease. *NAT GENET* 2015;47(10):1121-30. doi: 10.1038/ng.3396 pmid:263433872015-10-01].
3. R. Malik, G. Chauhan, M. Traylor, et al. Multiancestry genome-wide association study of 520,000 subjects identifies 32 loci associated with stroke and stroke subtypes. *NAT GENET* 2018;50(4):524-37. doi: 10.1038/s41588-018-0058-3 pmid:295313542018-04-01].
4. C. J. Willer, E. M. Schmidt, S. Sengupta, et al. Discovery and refinement of loci associated with lipid levels. *NAT GENET* 2013;45(11):1274-83. doi: 10.1038/ng.2797 pmid:240970682013-11-01].
5. M. Kanai, M. Akiyama, A. Takahashi, et al. Genetic analysis of quantitative traits in the Japanese population links cell types to complex human diseases. *NAT GENET* 2018;50(3):390-400. doi: 10.1038/s41588-018-0047-6 pmid:294030102018-03-01].
6. N. Soranzo, S. Sanna, E. Wheeler, et al. Common variants at 10 genomic loci influence hemoglobin A(1)(C) levels via glycemic and nonglycemic pathways. *DIABETES* 2010;59(12):3229-39. doi: 10.2337/db10-0502 pmid:208586832010-12-01].
7. J. Dupuis, C. Langenberg, I. Prokopenko, et al. New genetic loci implicated in fasting glucose homeostasis and their impact on type 2 diabetes risk. *NAT GENET* 2010;42(2):105-16. doi: 10.1038/ng.520 pmid:200818582010-02-01].

8. M. Steenman, O. Espitia, B. Maurel, et al. Identification of genomic differences among peripheral arterial beds in atherosclerotic and healthy arteries. *Sci Rep* 2018;8(1):3940. doi: 10.1038/s41598-018-22292-y pmid:295004192018-03-02].
9. Y. Doring, H. D. Manthey, M. Drechsler, et al. Auto-antigenic protein-DNA complexes stimulate plasmacytoid dendritic cells to promote atherosclerosis. *CIRCULATION* 2012;125(13):1673-83. doi: 10.1161/CIRCULATIONAHA.111.046755 pmid:223883242012-04-03].
10. B. Williams, G. Mancia, W. Spiering, et al. 2018 ESC/ESH Guidelines for the management of arterial hypertension. *EUR HEART J* 2018;39(33):3021-104. doi: 10.1093/eurheartj/ehy339 pmid:301655162018-09-01].
11. American Diabetes A. 2. Classification and Diagnosis of Diabetes: Standards of Medical Care in Diabetes-2021. *DIABETES CARE* 2021;44(Suppl 1):S15-33. doi: 10.2337/dc21-S002 pmid:33298413.[(c) 2020 by the American Diabetes Association.:*2021-01-01].
12. S. Burgess, Smith G. Davey, N. M. Davies, et al. Guidelines for performing Mendelian randomization investigations. *Wellcome Open Res* 2019;4:186. doi: 10.12688/wellcomeopenres.15555.2 pmid:32760811.[Copyright: (c) 2020 Burgess S et al.:*2019-01-20].
13. Baron RM, D. A. Kenny. The moderator-mediator variable distinction in social psychological research: conceptual, strategic, and statistical considerations. *J PERS SOC PSYCHOL* 1986;51(6):1173-82. doi: 10.1037//0022-3514.51.6.1173 pmid:38063541986-12-01].
